# Matrix Metalloproteinase 14 in the Zebrafish: An Eye on Retinal and Retinotectal Development

**DOI:** 10.1371/journal.pone.0052915

**Published:** 2013-01-09

**Authors:** Els Janssens, Djoere Gaublomme, Lies De Groef, Veerle M. Darras, Lut Arckens, Nathalie Delorme, Filip Claes, Inge Van Hove, Lieve Moons

**Affiliations:** 1 Research Group Neural Circuit Development and Regeneration, Department of Biology, Katholieke Universiteit Leuven (KU Leuven), Leuven, Belgium; 2 Laboratory of Comparative Endocrinology, Department of Biology, Katholieke Universiteit Leuven (KU Leuven), Leuven, Belgium; 3 Laboratory of Neuroplasticity and Neuroproteomics, Department of Biology, Katholieke Universiteit Leuven (KU Leuven), Leuven, Belgium; Institut Curie, France

## Abstract

**Background:**

Matrix metalloproteinases (MMPs) are members of the metzincin superfamily of proteinases that cleave structural elements of the extracellular matrix and many molecules involved in signal transduction. Although there is evidence that MMPs promote the proper development of retinotectal projections, the nature and working mechanisms of specific MMPs in retinal development remain to be elucidated. Here, we report a role for zebrafish Mmp14a, one of the two zebrafish paralogs of human MMP14, in retinal neurogenesis and retinotectal development.

**Results:**

Whole mount *in situ* hybridization and immunohistochemical stainings for Mmp14a in developing zebrafish embryos reveal expression in the optic tectum, in the optic nerve and in defined retinal cell populations, including retinal ganglion cells (RGCs). Furthermore, Mmp14a loss-of-function results in perturbed retinoblast cell cycle kinetics and consequently, in a delayed retinal neurogenesis, differentiation and lamination. These Mmp14a-dependent retinal defects lead to microphthalmia and a significantly reduced innervation of the optic tectum (OT) by RGC axons. Mmp14b, on the contrary, does not appear to alter retinal neurogenesis or OT innervation. As mammalian MMP14 is known to act as an efficient MMP2-activator, we also explored and found a functional link and a possible co-involvement of Mmp2 and Mmp14a in zebrafish retinotectal development.

**Conclusion:**

Both the Mmp14a expression in the developing visual system and the Mmp14a loss-of-function phenotype illustrate a critical role for Mmp14a activity in retinal and retinotectal development.

## Introduction

Retinal development is a tightly regulated process that requires coordinated cell proliferation, cell cycle exit and cell fate decisions [1]. The zebrafish eye is a powerful model system to study retinal and retinotectal pathway development because it is quite accessible and easy to manipulate, and its function and anatomy are fairly well understood and highly conserved in vertebrates [2].

Eye development in zebrafish embryos occurs rapidly, resulting in a functional visual system by 5 days post fertilization (dpf). At 10 hours post fertilization (hpf), the optic lobes evaginate from the diencephalon and 10 hours later (at 20 hpf), these neuroectodermal cells invaginate to form the eye cups, which further develop to form the neural retina, the pigment epithelium and the optic stalk [3], [4]. At 24 hpf, the lens detaches from the surface ectoderm which will further develop into the cornea, and between 24 hpf and 36 hpf, the neuroretina undergoes rapid proliferation and increases in size substantially [3], [5], [6].

In all vertebrates, retinal neurogenesis starts from a uniform population of neuroepithelial cells, which will generate seven major cell classes, each taking a well-defined position in the laminated retina [7]. In zebrafish, retinal neurogenesis is initiated around 27 hpf when the first differentiated retinal ganglion cells (RGCs) become apparent in the ventronasal retina. This differentiation process reaches the ventrotemporal border of the choroid fissure 10 hours later in a wave-like manner and is known as the first mitotic wave forming the RGC layer (RGCL). A second mitotic wave, which starts around 38 hpf, forms the inner nuclear layer (INL) and generates amacrine, horizontal and bipolar cells. Finally, the third mitotic wave, occurring around 48 hpf, creates the outer nuclear layer (ONL) which contains the photoreceptor cells. Müller glia are the last cell type to be generated [8], [9].

The development of the retinotectal pathway, containing the axons of the RGCs, starts around 34–35 hpf when the first RGC axons leave the eye through the ventral fissure and appear in the optic stalk. The axons of both eyes cross each other at the midline of the diencephalon to form the optic chiasm. Next, they bend dorsally to enter the contralateral optic tract and run straight to the optic tectum (OT), where they arrive around 46–48 hpf. Between 48 hpf and 72 hpf the RGC axons innervate the neuropil of the OT to form a very accurate topographic map of visual impulses within the brain [2], [10], [11].

Matrix metalloproteinases (MMPs) are members of the metzincin superfamily named after the zinc ion and the conserved Methionine residue at the active site [12]. These proteinases are mostly known for their ability to cleave and remodel the extracellular matrix, but they also (in)activate many signaling molecules such as growth factors, adhesion molecules and cytokines. In the nervous system, MMPs have been largely associated with disease onset and injury. However, there is mounting evidence that these proteinases may also regulate genesis, migration and survival of neurons in the developing brain [13]–[15]. *In vivo* exposed-brain experiments in developing *Xenopus* tadpoles revealed a requirement for MMPs in the proper development of the retinotectal projection by regulating the caudal turn towards the OT as well as its innervation. Administration of the broad-spectrum MMP inhibitor GM6001 onto exposed brain preparations of late tailbud stage *Xenopus* embryos altered the mid-diencephalic turning angle of RGC axons, that consequently failed to reach the anterior border of the OT. The use of a more specific MMP2/MMP9 inhibitor (SB-3CT) resulted in RGC axons that did make the caudal turn but failed to recognize the OT as their target of innervation [13], [16]. Furthermore, expression studies in different animal models also provide evidence for a role of MMPs in eye and retinal development. Indeed, *Dm2*-MMP is expressed in discrete regions of brain and eye imaginal discs of *Drosophila* larvae [17], MMP2 and MMP13 are expressed during chick corneal development [18] and MMP2, MMP9, and MMP14 are present in developing mouse retinas [19]. However, which specific MMPs play a role in retinal neurogenesis and/or retinotectal axon guidance still remains to be elucidated.

Here, we report a role for Mmp14a in retinal and retinotectal development in zebrafish larvae. Our results indicate that Mmp14a is necessary for proper retinoblast cell cycle kinetics, retinal neurogenesis, differentiation and lamination, and RGC axon innervation of the OT. Furthermore, we demonstrate a possible *in vivo* co-involvement of Mmp2 and Mmp14a in retinal and retinotectal development.

## Materials and Methods

### Zebrafish husbandry

Zebrafish (Danio rerio) were maintained at 28°C on a 14 h light/10 h dark cycle. Embryos were collected after natural spawning, staged as previously described [20], and raised at 28°C. Larvae older than 5 dpf were fed twice daily during the period of the experiment. Transgenic zebrafish expressing green fluorescent protein (GFP) in their RGCs, under control of the Ath5 promotor (*Tg(Ath5:EGFP)*), were provided by Dr. S. Wilson and described elsewhere [21]. All animal experiments were approved by the Institutional Ethical Committee of the KU Leuven and executed in strict accordance with the European Communities Council Directive of 24 November 1986 (86/609/EEC).

### Whole mount in situ hybridization (ISH), immunohistochemistry (IHC) and Alcian blue staining

Embryos at various developmental stages were fixed in 4% phosphate-buffered paraformaldehyde (PFA) overnight at 4°C, dehydrated in methanol and stored in methanol at −20°C. ISH was performed as previously described [22]. The sequences of primers used in synthesizing probes are: *mmp14a*-forward: 5′-TGGGTATCTTCCTCCTGGTG-3′ and *mmp14a*-reverse: 5′-GTTGTCCAAGGCTCTGCTTC-3′; *mmp14b*-forward: 5′-GGAATCAGAGCGGAAAATGA-3′ and *mmp14b*-reverse: 5′-AAAGCCATCAGCGAAGAAGA-3′; *mmp2*-forward: 5′-TTCTTGCTTCCCTGCAAACT-3′ and *mmp2*-reverse: 5′-GCTCCTGGATGCCTTTAACA-3′. The following anti-sense RNA probes were used: *otx2* [23] (kind gift from Dr. D. Zivcovic), *pax2.1*, *dlx2*, *shh* (kind gifts from Dr. G. David).

Embryos for whole mount immunostaining were fixed overnight in Dent's fixative (80% methanol with 20% DMSO). After 3 washes in PBST (PBS with 0.1% Tween-20), they were incubated for 1–2 h in prechilled aceton at −20°C and blocked in blocking solution (1% BSA, 1% DMSO in PBS) for 1 h at RT. Next, embryos were incubated in rabbit anti-acetylated-α-Tubulin primary antibody (Sigma, T9026, 1/1000 in PBS containing 1% DMSO), for 5 h at RT, followed by 3 times 20 min washes with blocking solution and overnight incubation at 4°C in an Alexa Fluor 568 goat-anti-mouse secondary antibody (Invitrogen, 1/200 in PBS containing 1% DMSO). The following day, the embryos were imaged after extensive washes with PBST.

Craniofacial cartilage elements were visualized by Alcian blue staining as previously described [24].

Whole mount images were acquired using a SteREO Discovery.V8 stereomicroscope from Zeiss. Fluorescent labeling was imaged using an Olympus FV1000 confocal microscope at 20× or 60× magnification. Images were adjusted for brightness and contrast using Adobe Photoshop CS5. For live imaging, *Tg(Ath5:EGFP)* embryos were treated with 0.003% of 1-phenyl-2-thiourea from 6 hpf onwards to prevent pigmentation.

### Immunohistochemistry (IHC) and histology on sections

For all histological and immunohistochemical stainings, embryos at various developmental stages were fixed in 4% PFA (unless otherwise mentioned) and processed for paraffin (sections of 7 µm) or cryo (sections of 10 µm) sectioning. Embryos stained by whole mount ISH were embedded in paraffin, sectioned and counterstained with Nuclear Fast Red (Sigma). Immunostaining for Mmp14a and Mmp2 was performed using rabbit primary antibodies against zebrafish Mmp14a (Anaspec, 55115, 1/100), mouse MMP14 (Abcam, ab53712, 1/200), zebrafish Mmp2 (Anaspec, 55111, 1/100) or mouse MMP2 (Santa Cruz, sc-8835-R, 1/200), followed by a biotinylated goat anti-rabbit secondary antibody (Dako, 1/300). Staining was obtained using horseradish peroxidase (HRP)-labeled streptavidin followed by amplification via the TSA™ FT/Cy3 system (Perkin Elmer). Additional fluorescent immunostainings were performed using appropriate Alexa Fluor secondary antibodies (Invitrogen) or the TSA™ FT/Cy3 System for the following primary antibodies: anti-BrdU (AbD serotec, OBT0030CX, 1/500), anti-Pax6 (Covance, PRB-278P, 1/300), anti-Pax6a (Anaspec, 55545, 1/200) and anti-Phospho-Histone H3 (Cell Signaling, 9701, 1/300). Anti-Zn5 (ZIRC, 1/100), anti-Zpr1 (ZIRC, 1/100) and anti-Zrf1 (ZIRC, 1/100) stainings were based on the protocol described by Uribe and Gross [25]. Imaging was performed using an Olympus FV1000 confocal microscope at 20× or 60× magnification. To generate semi-thin sections (1 µm), embryos were fixed in 2% glutaraldehyde, processed and stained with toluidine blue as previously described [26] and imaged using a Zeiss Imager Z1 microscope at 20×. Images were adjusted for brightness and contrast using Adobe Photoshop CS5.

### Compound experiments

To block overall MMP activity, embryos were treated with the broad-spectrum MMP inhibitor GM6001 (Santa Cruz, sc-203979) at 200 µM or with EDTA, a broad-spectrum metalloproteinase inhibitor, at 500 µM from 30 hpf onwards (*n* = 30 from 3 independent experiments).

### Microinjections of antisense morpholino

Morpholinos (MO) were obtained from Gene Tools, LLC: *mmp14a*-ATG-MO: 5′-GACGGTACTCAAGTCGGGACACAAA-3′ [27]; *mmp14a-*splice-MO: 5′-TAAGACTGGGCGAGACTTACGAGAG-3′ [27]; *mmp14b*-ATG-MO: 5′-AAACCCGCTCCAGATCATTTTCCGC-3′ [28]; *mmp14b*-splice-MO: 5′-AATGCATGATACTCACCTCAGGTTT-3′; Standard control-MO: 5′-CCTCTTACCTCAGTTACAATTTATA-3′; *p53*-MO: 5′-GACCTCCTCTCCACTAAACTACGAT-3′ [29]; *mmp2*-ATG-MO: 5′-ATCTGAAAAACTTAACGGACAGCAT-3′. After defining the optimal working concentrations via dose-response experiments, embryos were injected with the optimal doses of MO into the yolk at the 1- to 4-cell stage. The Mmp14a-ATG MO, the standard control MO, the p53 MO and Mmp14b-ATG MO were injected at a concentration of 2 ng, the Mmp14a-splice MO was used at 4 and 9 ng, the Mmp14b-splice MO was used at 9 and 13 ng, while the Mmp2-ATG MO was used at 1 to 4 ng. Knockdown efficiency was verified by Western blotting for the ATG MOs (see below) and via RT-PCR for the splice MOs, using the following primers: *mmp14a*-splice-forward (F): 5′-TGGGTATCTTCCTCCTGGTG-3′; *mmp14a*-splice-reverse (R): 5′-TTATCAGGAACGCCACATCTC-3′; *mmp14b*-splice-forward (F): 5′- GGAATCAGAGCGGAAAATGA-3′; *mmp14b*-splice-reverse 1 (R1): 5′-AGCGCTTCTTTCTCAGGTTG-3′; *mmp14b*-splice-reverse 2 (R2): 5′- TGCCTTTTAGCACACACACTT-3′, β-actin-forward (F): 5′- GCTACAGCTTCACCACCACA-3′ [27]; β-actin-reverse (R): 5′- CTTCTGCATACGGTCAGCAA-3′ [27].

The Mmp14b-splice MO was designed to target the exon 1/intron 1 junction of zebrafish *mmp14b*, thereby blocking the splice donor site and preventing correct splicing of intron 1. Since intron 1 is >11 kb, Mmp14b-splice MO efficiency was validated using two separate primer sets, one generating a fragment spanning exon 1 to 3 (F-R1) to detect correctly spliced transcripts, and one generating a fragment that spans exon 1 and part of intron 1 (F-R2) to detect aberrantly spliced transcripts. As loading control, a house-keeping gene (β-actin) was amplified.

### Western blotting

Embryos of 30 hpf were homogenized in lysis buffer (10 mM Tris-HCl pH8, 1% Triton X-100, 150 mM NaCl, 0.1% SDS, 0.5% NaDOC, 0.2% Na-azide) supplemented with protease inhibitors. Western blot analysis was performed as described in Van Hove *et al* [30]. The same primary antibodies as for IHC were used: anti-Mmp14a (Anaspec, 55115, 1/250), anti-MMP14 (Abcam, ab53712, 1/500), anti-Mmp2 (Anaspec, 55111, 1/250) and anti-MMP2 (Santa Cruz, sc-8835-R, 1/200). Western blots showed that the antibodies raised against mouse or zebrafish MMPs labeled similar bands representing either zebrafish Mmp14a or zebrafish Mmp2 (see Results section and data not shown). Subsequent coomassie blue staining of the PVDF membranes was used as loading control [31].

### Examination of early developmental defects

Examination of gastrulation defects was performed by scoring stage of development at tail bud stage using a semi-quantitative scoring system. The embryos at a correct tail bud stage received a score of “normal” while embryos with a delayed development received a score of “class I” for 80% epiboly stage or “class II” for 50% epiboly stage (*n* = 141 from 3 independent experiments).

The otic vesicle length (OVL) was analysed at 31 hpf as previously described [20], [32]. The OVL is obtained by dividing the ear-eye-distance by the inner ear diameter (OVL = EED/IED), with the highest OVL corresponding to the least developed embryo (*n* = 86 from 2 independent experiments).

### BrdU labeling and acridine orange staining in zebrafish embryos

BrdU incorporation assays were performed by incubating embryos at several developmental stages for 30 min in 10 mM BrdU solution (in fish medium with 15% DMSO) on ice. Afterwards, the embryos were thoroughly washed, placed back at 28°C for 1 h and fixed for BrdU immunostaining. Midsagittal sections were photographed at the level of the optic nerve using an Olympus FV1000 confocal microscope at 60× magnification. Analysis at 72 hpf was performed on 5 embryos per condition by counting all BrdU^+^ cells on 3 sections per retina and averaging towards the number of BrdU^+^ cells/section (*n* = 15 from 3 independent experiments).

To determine the time needed for retinoblasts to proceed from S to M phase the percent labeled mitosis (PLM) paradigm test was used as described [33]. Embryos, exposed to a 15 min BrdU pulse at 31 hpf, were fixed every 15 min for the first hour and every 30 min between 1 and 2.5 h after exposure, and subsequently embedded in paraffin and sectioned. Double immunostainings for Phospho-Histone H3 (PH3) and BrdU were used to reveal the number of cells in M-phase (PH3^+^) and in S-phase (BrdU^+^) at the time of fixation. Cells that were in the S phase at BrdU exposure and are PH3^+^/BrdU^+^ at the time of fixation are those that have progressed from S to M phase. The PLM represents the proportion of PH3^+^ cells that are also BrdU^+^ and graphing this percentage over time gives an estimation of the duration to switch from S to M phase. Analysis was performed as above on 5 embryos per condition by counting all BrdU^+/^PH3^+^ cells on 3 sections per retina (*n* = 10 from 2 independent experiments).

To detect apoptosis, embryos at 32, 48 and 72 hpf were placed in embryo medium containing 2 µg/ml of acridine orange for 30 min, rinsed 8 times 5 min with embryo medium and photographed with an Olympus FV1000 confocal microscope at 20× magnification (*n* = 20 from 4 independent experiments).

### Quantification of retinal neurogenesis and tectal innervation

Analysis of retinal neurogenesis wave defects was performed by semi-quantitative scoring of the stage of neurogenesis at 49 hpf. Embryos showing only 25% completion of the neurogenesis wave were scored as “25%” while embryos with a normal and completed neurogenesis received a score of “100%” (*n* = 45 from 3 independent experiments).

Eye diameter and body (head-tail) length were determined after photographing living embryos with a SteREO Discovery.V8 Zeiss stereomicroscope up to 11 dpf. The embryos were anesthetized with 0.05% Tricaine and carefully positioned on their side in agar lanes. All eye size measurements were performed along the anteroposterior axis.

To quantify the tectal innervation area in embryonic brains, acetylated-α-Tubulin stained or *Tg(Ath5:GFP)* embryos were imaged with a SteREO Discovery.V8 Zeiss stereomicroscope. The embryos were placed in agar lanes in a defined position to obtain a dorsolateral view in order to nicely expose the OT. The Tubulin- or Ath5-positive area of the neuropil was analysed by calculating the pixel area according to previously published methods [34]. All analyses were performed using the digital imaging processing software Axiovision 4.7 (Zeiss).

Eye diameter, body length and tectal innervation area were normalized towards average values from control fish when comparing different doses of MO at one developmental stage. However, when comparing different developmental stages, absolute values were provided to enable visualizing embryo growth. Between 15 and 30 injected embryos were analysed per experiment to identify alterations in eye diameter, body length or tectal innervation area, and each experiment was repeated at least three times, unless otherwise mentioned.

### Statistics

For the gastrulation scoring, data were analysed using Chi-square statistics. For analysis of eye size, body length and OT innervation, a multilevel model (SAS proc mixed) was used to compare various developmental stages, while for all other statistical analyses a two-tailed Student's *t*-test was applied on normalized data unless otherwise mentioned. A *p* value of 0.05 was considered statistically significant. All values are represented as mean ± SEM.

## Results

### Spatiotemporal expression of Mmp14 in the developing zebrafish

**Figure 1:**
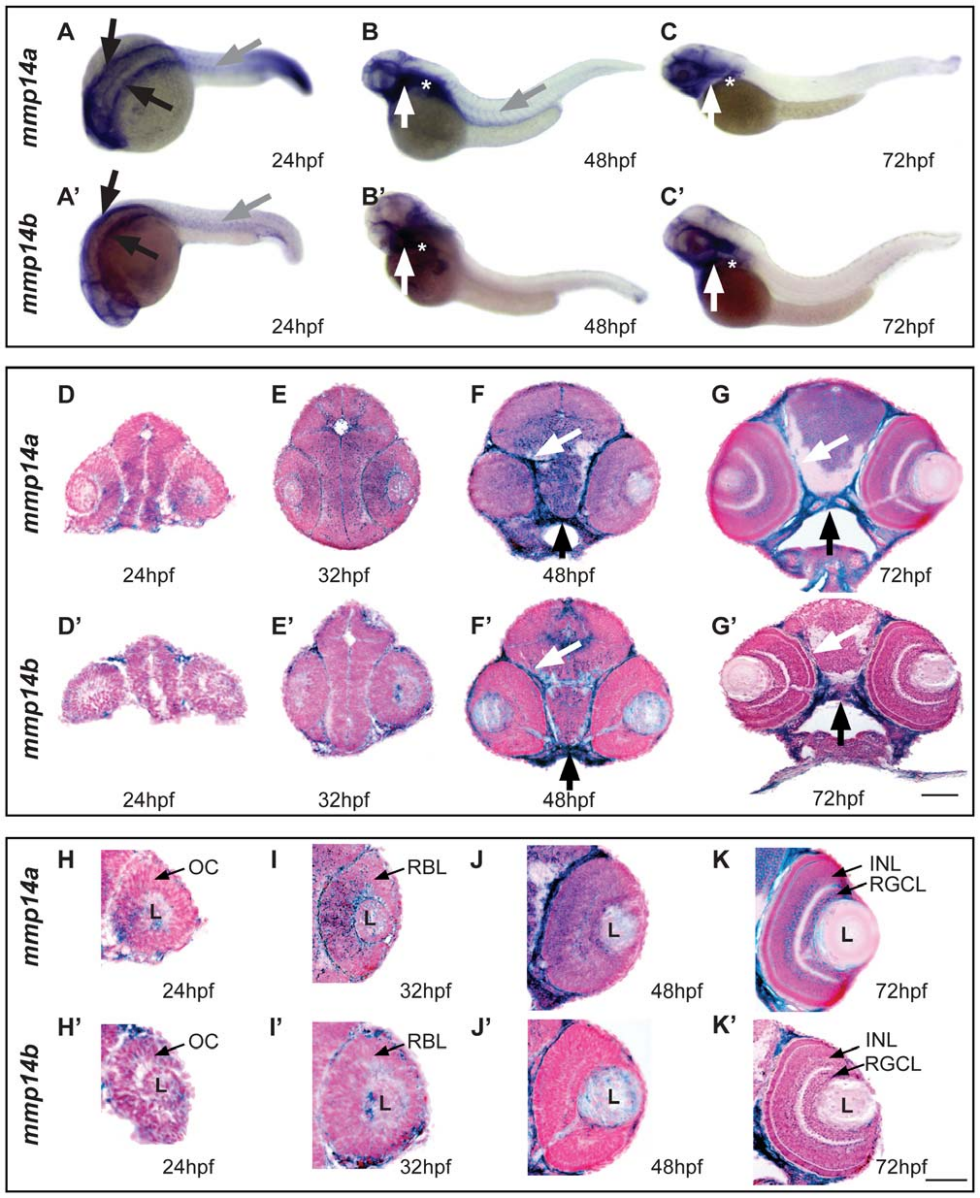
Spatiotemporal expression pattern of *mmp14a* and *mmp14b* mRNA in developing zebrafish. **A–C & A′–C′** Whole mount *in situ* hybridization (ISH) for *mmp14a* (**A–C**) and *mmp14b* (**A′–C′**) in zebrafish embryos at various developmental stages shows *mmp14a* and *mmp14b* expression in the head mesenchyme lateral and ventro-lateral to the hindbrain (black arrows in **A** and **A′**) at 24 hpf, as well as in myosepta in the trunk and tail (grey arrow in **A,** **B** and **A′**) at 24 hpf and 48 hpf. *Mmp14a* and *mmp14b* expression in craniofacial elements (white arrow in **B–C, B′–C′**) and pectoral fins (white star in **B–C, B′–C′**) is apparent from 48 hpf onwards. **D–G & D′–G′** Transverse sections through the head of zebrafish embryos, stained via whole mount ISH for *mmp14a* (**D–G**) and *mmp14b* (**D′–G′**) and counterstained with Nuclear Fast Red, show mRNA expression in the brain, cartilage (black arrow) and connective tissue (white arrow) at various developmental stages. **H–K & H′–K′** Detailed view of the retina at various developmental stages shows prominent *mmp14a* expression in the optic cup (**H**) at 24 hpf, the retinoblast layer (**I, J**) at 32 and 48 hpf and in the INL and RGCL (**K**) at 72 hpf, while *mmp14b* expression in the retina is almost absent (**H′–K′**). hpf, hours post fertilization; INL, inner nuclear layer; L, lens; OC, optic cup; RBL, retinoblast layer; RGCL, retinal ganglion cell layer. Scale bars: 50 µm.

Zebrafish possess two Mmp14 paralogs, *mmp14a* and *mmp14b*, which are known to be expressed in overlapping regions during embryonic development from the one-cell stage to 5 days post fertilization (dpf), with high expression in the head region, pectoral fins and craniofacial cartilage elements [27], [28]. Whole mount *in situ* hybridization (ISH) indeed revealed both *mmp14a* and *mmp14b* mRNA in the head mesenchyme, lateral and ventro-lateral to the hindbrain at 24 hpf (Figure 1A, 1A′). *Mmp14a* expression was also detected in the myosepta at 24 hpf and 48 hpf (Figure 1A, 1B), while this was only visible at 24 hpf for *mmp14b* (Figure 1A′). At 48 and 72 hpf, prominent expression of *mmp14a* and *mmp14b* was found in the head, including craniofacial elements, and in the pectoral fins (Figure 1B, 1B′, 1C, 1C′). Subsequent sectioning of whole mount labeled embryos at different developmental stages revealed prominent *mmp14a* and *mmp14b* expression in the perichondria of the cartilage elements within the pharyngeal arches as well as in the connective tissue surrounding the brain, the nasal pit and the eye and a moderate expression in the brain (Figure 1D–G and 1D′–G′). Moreover, *mmp14a* was clearly expressed in the eye cup as early as 24 hpf, in the retinoblast layer at 32 hpf and became restricted to the retinal ganglion cell layer (RGCL) and the inner nuclear layer (INL) at 72 hpf (Figure 1H–K). On the contrary, only low *mmp14b* expression was found in the retina at all developmental stages (Figure 1H′–K′).

**Figure 2:**
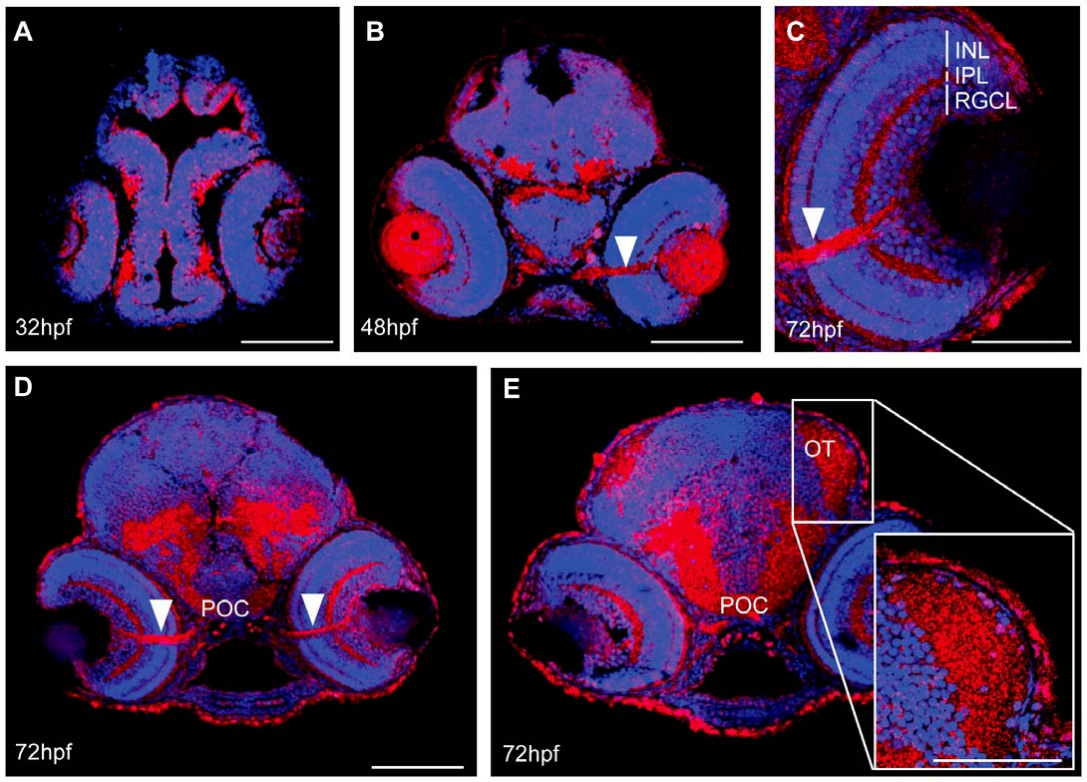
Spatiotemporal expression pattern of MMP14 protein in developing zebrafish. **A–E** Immunohistochemical stainings on transverse sections, made through the head of zebrafish embryos of various developmental stages show MMP14 expression (red) in the retina and the brain from 32 hpf onwards (**A**). At 48 hpf and 72 hpf (**B–D**) expression is visible in the optic nerve (white arrowhead), in the POC and in the neuropil of the OT (**D**, **E**). Higher magnification of the eye at 72hpf (**C**) reveals MMP14 expression in the INL, IPL and RGCL as well as in the optic nerve. The inset in **E** gives a detailed view of the MMP14 positive tectal neuropil. DAPI (blue) was used as counterstain. hpf, hours post fertilization; INL, inner nuclear layer; IPL, inner plexiform layer; OT, optic tectum; POC, postoptic commissure; RGCL, retinal ganglion cell layer. Scale bars: 50 µm, except inset in panel E: 100 µm.

Immunohistochemistry with an antibody against mouse MMP14 confirmed Mmp14 protein expression in the retina and brain at 32, 48 and 72 hpf (Figure 2A, 2B, 2D, 2E). At 72 hpf, a pronounced Mmp14 expression was observed in the inner plexiform layer (IPL), the INL and RGCL of the retina (Figure 2C, 2D), in the optic nerve and in the postoptic commissure (POC) (Figure 2C, 2D) as well as in the neuropil of the OT (Figure 2E). Similar stainings were obtained using an antibody against zebrafish Mmp14a (data not shown).

Together, these expression data clearly reveal Mmp14a expression in the developing retina and retinotectal projection areas, suggestive for its involvement in visual system development. Mmp14b expression appears to be largely confined to brain regions, connective tissue and cartilage elements.

### Mmps are involved in development of the zebrafish retinotectal pathway

**Figure 3:**
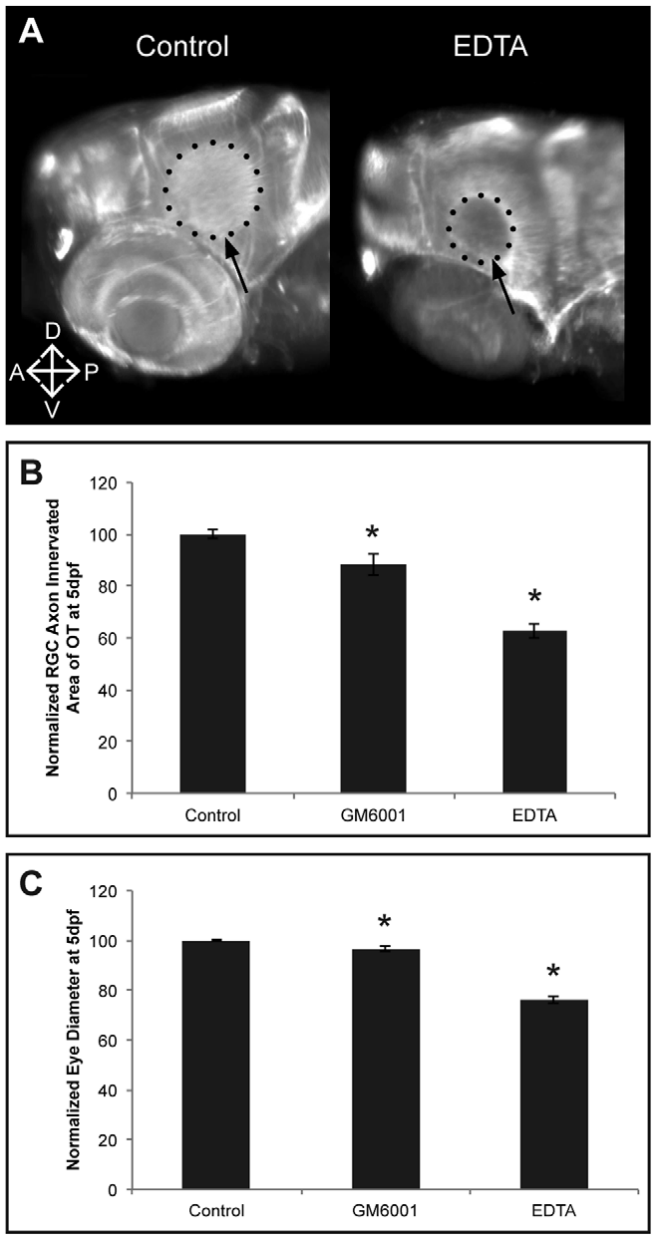
EDTA/GM6001 treatment results in microphthalmic embryos showing reduced RGC axon innervation in the optic tectum. **A** Whole mount immunostaining for acetylated-α-Tubulin in 5 dpf EDTA-treated embryos reveals a reduced tectal area (dotted circle and arrow) innervated by RGC axons, represented by the smaller dotted circle in the EDTA-treated embryos as compared to control embryos. Images show a dorsolateral view on the left OT and eye. **B–C** Quantitative analysis of the tectal area innervated by RGC axons and the eye size reveals that EDTA as well as GM6001 treatment leads to embryos with a significantly reduced RGC axon arborization area and microphthalmic eyes, as compared to control embryos at 5 dpf. Eye size and tectal innervation area are normalized to average values in untreated embryos (*n* = 30 from 3 independent experiments). Data are represented as mean ± SEM (*p<0.05, Student's *t*-test). A; anterior; D, dorsal; dpf: days post fertilization; OT: optic tectum; P, posterior; RGC: retinal ganglion cells; V, ventral.

As Mmp14a is expressed in the developing retina and midbrain and as MMPs are known to interfere in major decision points during retinotectal pathway development in *Xenopus* [13], [16], we investigated a possible contribution of Mmps to the development of the retinotectal pathway in zebrafish. Therefore, EDTA, a broad-spectrum metalloproteinase inhibitor, was administered to the embryo medium at 30 hpf. Anti-acetylated-α-Tubulin staining on EDTA-treated embryos revealed a reduced OT innervation area by RGC axons despite an overall normal morphology at 5 dpf (Figure 3A, 3B). Moreover, the eye diameter was significantly reduced in EDTA-treated embryos, as compared to controls (Figure 3A, 3C). Similar, although milder, results were found using the broad-spectrum MMP inhibitor, GM6001, at 30 hpf (Figure 3B, 3C). These findings suggest a role for Mmps in zebrafish eye and retinotectal development.

### Mmp14a knockdown does not affect gastrulation and overall embryonic development

**Figure 4:**
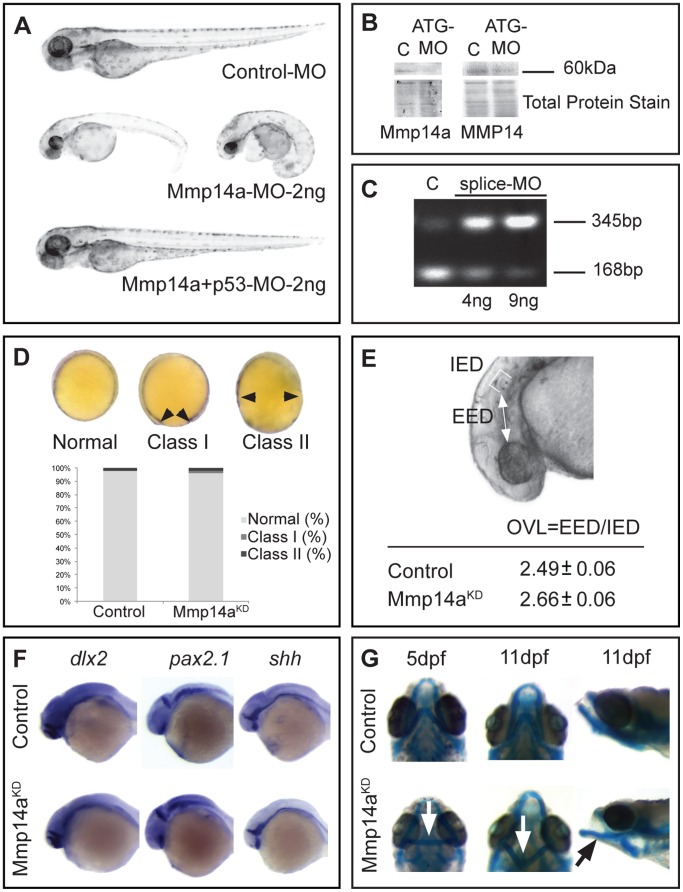
Mmp14a knockdown minimally affects overall embryonic morphogenesis. **A** Knockdown of Mmp14a with 2 ng of Mmp14a-ATG MO at 3 dpf results in embryos with edema and a deformed body axis. However, embryos injected with 2 ng of both Mmp14a-ATG and p53 MOs do not show any morphological abnormalities. **B** Validation of Mmp14a-ATG MO efficiency via Western blotting using antibodies to zebrafish Mmp14a or to mouse MMP14, reveals reduced levels of active Mmp14a protein (60 kDa band) in 30 hpf Mmp14a morphants. Total protein coomassie blue staining was used for loading control. **C** RT-PCR analysis of Mmp14a-splice MO injected embryos reveals efficient splice blocking, resulting in a dose-dependent increase in aberrantly spliced *mmp14a* mRNA (345 bp band) and a concomitant decrease in correctly spliced *mmp14a* mRNA (168 bp band). **D** Semi-quantitative scoring of gastrulation defects at the tail bud stage (10 hpf) does not reveal a significant delay in gastrulation in Mmp14a morphant (Mmp14a^KD^) embryos, as compared to control embryos. The black arrowheads indicate the position of the germ cell layer (*n* = 141 from 3 independent experiments). **E** Measurements of the OVL, analysed by dividing EED by IED, in 31 hpf embryos, confirms the absence of a developmental delay after Mmp14a knockdown (*n* = 86 from 2 independent experiments). **F** Whole mount *in situ* hybridization for the neural markers *dlx2*, *pax2.1* and *shh* shows normal development of the brain in control and Mmp14a morphant embryos at 32 hpf (*n* = 20 from 2 independent experiments). **G** Combined knockdown of Mmp14a and p53 (Mmp14a^KD^) only results in minor craniofacial abnormalities, including mild malformations of the Ceratohyal cartilage (white arrow) and the Meckel's cartilage (black arrow) at 5 and 11 dpf. Left and middle panel are ventral views, the right panel is a lateral view. C, control; dpf, days post fertilization; EED, Eye-Ear-Diameter; IED, Inner-Ear-Diameter; hpf, hours post fertilization; MO, morpholino; OVL, Otic Vesicle Length.

To investigate a possible role for Mmp14a in retinal and retinotectal development, morpholino (MO) knockdown technology was used. Injection of various concentrations of the previously published ATG- or splice Mmp14a MOs [27] consistently resulted in a high number of embryos showing edema, curled tails and a reduced body length. As MOs can sequence-independently activate the p53 apoptosis pathway [29], [35], co-injections with a p53 MO were performed and these resulted in morphants with an overall normal embryonic development (Figure 4A). The maximally tolerated dose of MO resulting in normally developing embryos, was determined at 2 ng for the Mmp14a-ATG MO and at 9 ng for the Mmp14a-splice MO, both when co-injected with 2 ng of p53 MO. The efficacy of the obtained Mmp14a knockdown was validated via Western blotting for the ATG-MO (Figure 4B) and by RT-PCR for the splice-MO (Figure 4C).

Since previously published data reported gastrulation defects after Mmp14a knockdown [27], the embryos co-injected with the Mmp14a-ATG MO or Mmp14a-splice MO and p53 MO were subjected to a semi-quantitative gastrulation scoring system. No significant gastrulation defects were found in any of the embryos after these combined injections with Mmp14a-ATG MO (Figure 4D) or Mmp14a-splice MO (data not shown). In all following experiments, combined injections with Mmp14a-ATG and p53 MO (from now on referred to as Mmp14a-ATG MO injected embryos) were used and compared to embryos co-injected with standard control MO and p53 MO (from now on referred to as control embryos) at MO concentrations of 2 ng, unless otherwise mentioned.

Next, Mmp14a knockdown embryos were subjected to a series of tests to further investigate embryonic development. Morphometric analysis of the otic vesicle length (OVL) at 31 hpf [20], [32] revealed no obvious developmental abnormalities in the Mmp14a morphants as compared to control MO injected embryos (Figure 4E). Importantly, also the expression of the neural markers *dlx2*, *pax2.1* and *shh* showed a normal brain development in Mmp14a-ATG MO injected embryos at 32 hpf (Figure 4F). Indeed, *dlx2* expression found medially in the telencephalon and the diencephalon [36], *pax2.1* expression in the optic stalk, the midbrain-hindbrain boundary [37] and the rhombomeres of the hindbrain, as well as *shh* expression in the optic stalk and the floor plate [37], were all similar in Mmp14a morphants as compared to control embryos.

Previously published data reported craniofacial dysmorphisms in MMP14 deficient mice and severely disrupted formation of the pharyngeal skeleton in zebrafish embryos after Mmp14a knockdown [27], [38]. However, at the ATG-MO concentration (2 ng) used in this study, combined with the p53 MO, Alcian blue staining revealed only mild craniofacial defects (malformations of Ceratohyal cartilage and Meckel's cartilage) in Mmp14a knockdown embryos at 5 and 11 dpf (Figure 4G).

Overall, detailed analyses reveal that the Mmp14a morphants develop normally without any non-specific MO-induced developmental delay.

### Mmp14a knockdown results in microphthalmia

**Figure 5:**
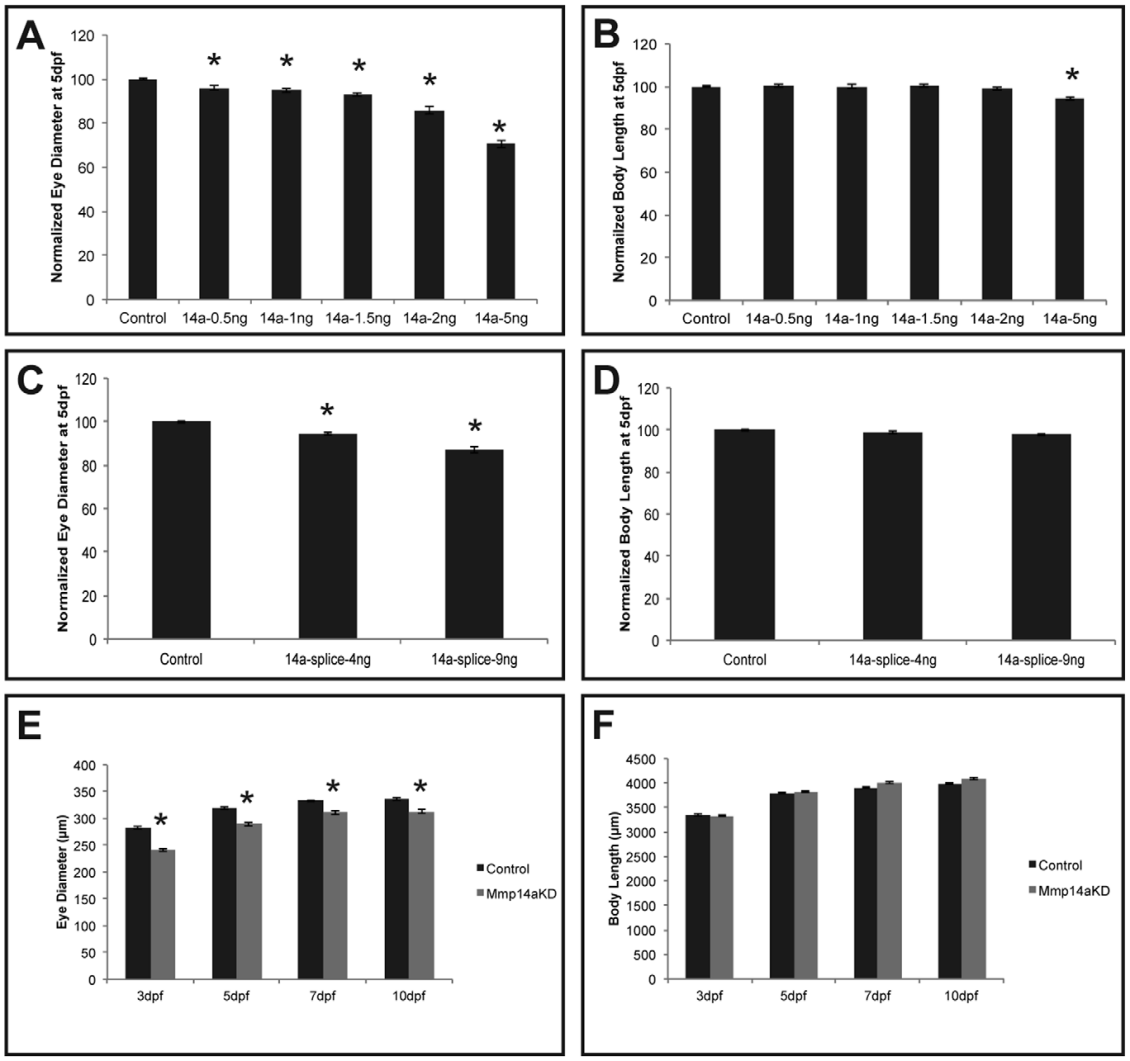
Mmp14a knockdown results in embryos with microphthalmic eyes. **A–D** Analysis of eye size (**A, C**) and total body length (**B, D**) reveals a dose-dependent decrease in eye size after knockdown of Mmp14a with the ATG- (**A, B**) or the splice MO (**C, D**) at 5 dpf. Body length, however, was only decreased after injection of a supramaximal dose of the Mmp14a-ATG MO (**B**) (*n* = 55 from 3 independent experiments). **E, F** Analysis of eye size (**E**) and total body length (**F**) (both in µm), in embryos injected with Mmp14a-ATG MO (Mmp14a^KD^), reveals that knockdown of Mmp14a leads to persistent microphthalmic eyes in embryos/larvae from 3 to 10 dpf while the overall body length is unaffected as compared to control fish (*n* = 75 from 3 independent experiments). Data are represented as mean ± SEM (*p<0.05, Student's *t*-test for **A–D**; multilevel model statistical test (SAS proc mixed) for **E–F**).

As our expression data suggest an involvement of Mmp14a in visual system development, we first looked at eye size. Quantification of the eye diameter revealed significantly smaller eyes in Mmp14a morphants. Indeed, dose-response experiments with the Mmp14a-ATG MO showed a dose-dependency of the observed phenotype (Figure 5A, 5B) and similar results were obtained using the Mmp14a-splice MO (Figure 5C, 5D). Higher concentrations of Mmp14a-ATG MO resulted in a more severe phenotype including smaller body sizes (Figure 5A, 5B). At optimal Mmp14a knockdown, the Mmp14a morphant eyes grew substantially between 3 and 5 dpf, but the eyes remained smaller than those of control embryos even up to 10 dpf, despite similar body lengths (Figure 5E, 5F).

In summary, Mmp14a knockdown results in microphthalmic embryos.

### Mmp14a is essential for retinal cell differentiation and lamination

**Figure 6:**
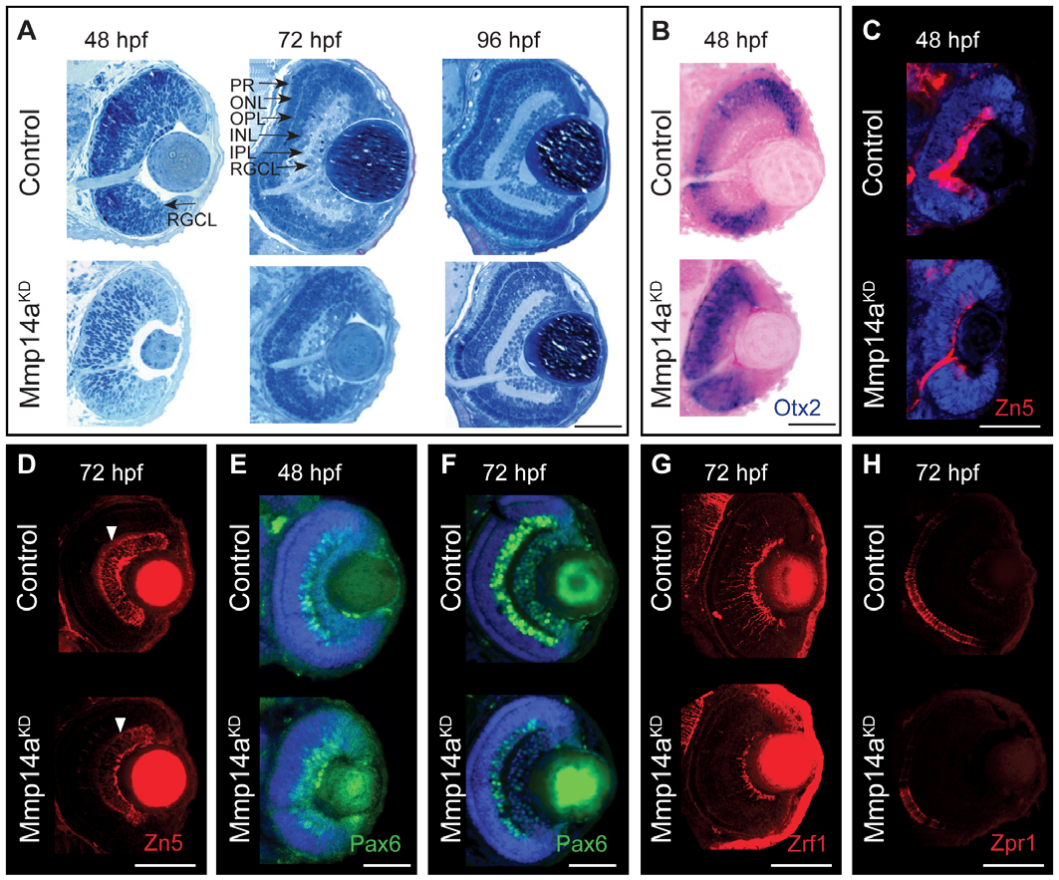
Mmp14a is required for timely differentiation of retinal neurons. **A** Toluidine blue stained transverse semi-thin sections show a delay in retinal differentiation and lamination in Mmp14a morphant eyes (Mmp14a^KD^). At 48 hpf, control embryos show an initial laminated retina, which develops into a fully laminated and differentiated retina at 72 hpf, with a clearly distinguishable RGCL, IPL, INL, OPL, ONL, and PR. Mmp14a morphant retinas, however, completely lack lamination at 48 hpf, while at 72 hpf the degree of lamination is still severely reduced, as compared to control embryos. At 96 hpf, however, morphant retinas appear fully laminated and differentiated and are indistinguishable from control retinas. **B** *In situ* hybridization for *otx2* shows increased expression in the central retina of transverse eye sections of morphant embryos as compared to controls. Nuclear Fast Red was used as counterstain. **C–H** Immunohistochemical stainings for markers of neuronal differentiation on transverse sections of Mmp14a morphant and control embryos reveal a reduced expression of Zn5 (red), which stains RGCs and their axons, in Mmp14a morphants at 48 hpf (**C**) and 72 hpf (**D**, white arrowhead shows RGC dendrites), of Pax6 (green), which labels RGCs and amacrine cells, at 48 hpf (**E**) and 72 hpf (**F**), of the Müller glia marker Zrf1 (red) (**G**), and of the photoreceptor marker Zpr1 (red) (**H**), both at 72 hpf, all indicative for a disrupted retinal differentiation. DAPI (blue) was used as counterstain in **C**, **E**, **F**. The images in panel **D**, **G** and **H** were taken using confocal microscopy. hpf, hours post fertilization; INL, inner nuclear layer; IPL, inner plexiform layer; ONL, outer nuclear layer; OPL, outer plexiform layer; PR: photoreceptor layer; RGCL, retinal ganglion cell layer. Scale bars: 50 µm.

Additional histological and immunohistochemical stainings were performed to analyze retinal development in more detail. Semi-thin sections of control retinas of 48 hpf showed a retina with a clear distinguishable RGCL, which developed into a fully differentiated retina with a morphologically distinct RGCL, INL and ONL at 72 hpf (Figure 6A). In contrast, in retinas of Mmp14a morphant embryos, lamination was barely detectable at 48 hpf and remained poorly developed at 72 hpf. Clearly distinguishable retinal layers only became visible in Mmp14a morphant embryos at 96 hpf (Figure 6A). In addition, the optic nerve was only rudimentary developed in morphant eyes at 48 hpf (Figure 6A).

To better reveal any changes in retinal differentiation due to Mmp14a knockdown, ISH and IHC for a panel of markers were performed on transverse eye sections of control and Mmp14a morphant embryos at 48 and/or 72 hpf. In control embryos of 48 hpf, *otx2*, a marker for postmitotic neuroblasts that will eventually differentiate into the various cell types of the neural retina [39], [40], was highly expressed in the INL and photoreceptor layer, both still differentiating at this time point. In Mmp14a morphants of 48 hpf, *otx2* expression appeared uniformly spread throughout all cells of the central retina, thereby indicating a delayed or disrupted retinal differentiation (Figure 6B) [41]. In addition, immunostaining for Zn5, labeling mature RGCs and their axons, showed a disturbed RGC differentiation at 48 hpf in the morphants and clearly revealed a reduced number of ganglion cell bodies and dendrites at 72 hpf (Figure 6C, 6D). High levels of Pax6 expression could be detected in RGCs and amacrine cells at 48 and 72 hpf in control retinas (Figure 6E, 6F). However, in Mmp14a morphant retinas, Pax6^+^ amacrine cells seemed to be missing at 48hpf, while a clearly diminished expression of Pax6 was observed at 72 hpf (Figure 6E, 6F). Moreover, also immunostainings for Zrf1, labeling radial fibers of Müller glia, and Zpr1, a cone-specific photoreceptor marker, revealed a disrupted retinal differentiation and lamination process at 72 hpf (Figure 6G, 6H). Of note, and possibly in part due to dilution and functional loss of Mmp14a MO, retinal lamination did catch up in the Mmp14a morphants, as at 96 hpf all retinal layers were formed (Figure 6A).

Thus, Mmp14a loss-of-function delays differentiation of the various retinal cell types, however, when retinal cells finally differentiate, they are correctly distributed within appropriate laminae.

### Mmp14a knockdown does not affect early eye development

**Figure 7:**
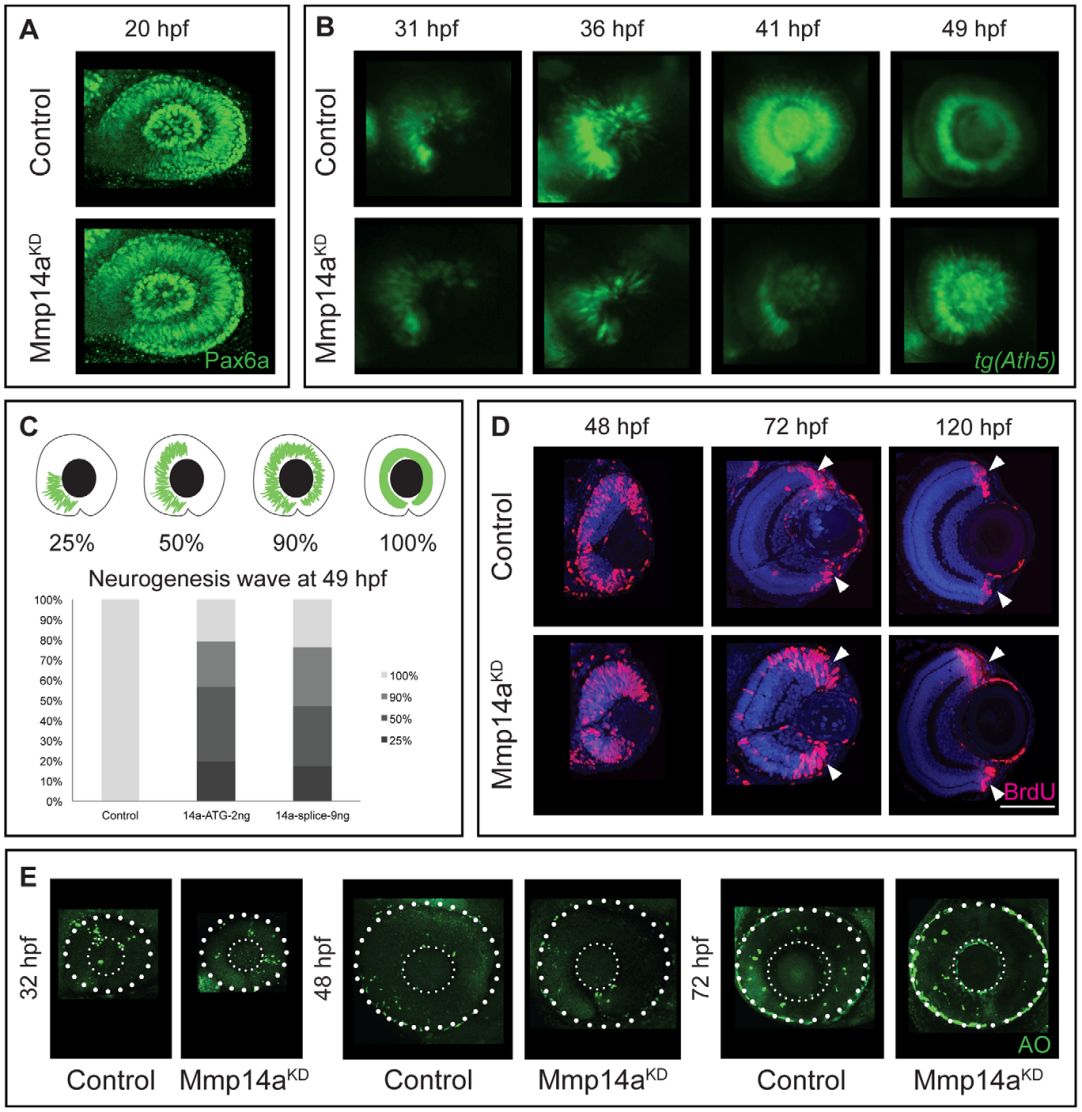
Mmp14a knockdown affects neurogenesis in the retina. **A** Whole mount immunostaining for Pax6a does not reveal any differences in eye morphology and Pax6a expression between Mmp14a morphant and control retinas at 20 hpf. **B** Live imaging of retinal neurogenesis in transgenic *Tg(Ath5:GFP)* embryos shows a small patch of Ath5^+^ cells in the ventronasal region of the developing eye in control embryos at 31 hpf. The Ath5^+^ cell population expands in a wave and reaches the ventrotemporal region of the retina around 41 hpf. Mmp14a morphant eyes show a delay in neurogenesis and the Ath5^+^ cell population only reaches the ventrotemporal region of the retina around 49 hpf. **C** The progression of the expansion of the Ath5^+^ cell population at 49 hpf is semi-quantitatively scored as illustrated in the top scheme. Injection of both the Mmp14a-ATG and the Mmp14a-splice MOs resulted in a severe delay of the neurogenesis wave (*n* = 45 from 3 independent experiments). **D** BrdU incorporation assays, performed at various developmental stages, show a higher number of immunopositive BrdU (BrdU^+^) cells in transverse sections of morphant retinas at 48 hpf. At 72 hpf, the proliferating region is restricted to the CMZ (white arrowhead) in control retinas, whereas BrdU^+^ cells are still abundantly detected in the central retina of Mmp14a morphant eyes. Even at 120 hpf, the proliferating region in morphant retinas is less confined to the CMZ as compared to control retinas. DAPI (blue) was used as counterstain. **E** Labeling of apoptosis using the fluorescent marker acridine orange does not reveal significant differences in the number of apoptotic cells at various developmental stages between Mmp14a morphant and control retinas. The small dotted circle marks the lens, the larger circle lines the eye (*n* = 20 from 4 independent experiments). AO, acridine orange; CMZ, cilliary marginal zone; hpf, hours post fertilization. Scale bar in D: 50 µm.

As the small eye phenotype and the delayed differentiation could be due to effects of Mmp14a knockdown on early eye developmental processes, early eye development was analyzed using immunostaining for Pax6a, a transcription factor known to be essential for transformation of optic vesicles into eyes between 16–24 hpf [42]. When comparing control and morphant eye cups at 20 hpf, no difference in overall morphology and in Pax6a expression was observed (Figure 7A), indicating that knockdown of Mmp14a does not affect early eye development.

### Mmp14a knockdown affects neurogenesis in the retina

Microphthalmic eyes could also be the result of disturbed retinal neurogenesis. Retinal neurogenesis begins when ganglion cell precursors become postmitotic in a cluster of Ath5^+^ ventronasal cells between 27 and 28 hpf. These Ath5^+^ cells migrate in a wave-like manner and ultimately reach the ventrotemporal border of the choroid fissure 10 h later [9], [42]. To study this process, we took advantage of the *Tg(Ath5:EGFP)* line [21], which allows live imaging of the neurogenesis wave at different stages of development. In control embryos, retinal neurogenesis started around 27 hpf and was visible as a small patch of Ath5^+^ cells in the ventronasal retina. These cells moved like a fan to reach the ventrotemporal region around 41 hpf (Figure 7B). The first Ath5^+^ cells only became visible in Mmp14a morphants at 31 hpf and retinal neurogenesis was clearly delayed, as 80% of the morphant embryos failed to complete this process at 49 hpf (Figure 7B and 7C). Similar results were found with the Mmp14a-splice MO (Figure 7C).

To further investigate the delay in retinal neurogenesis, BrdU pulse experiments were performed at various embryonic stages (Figure 7D). In control embryos, the number of proliferating BrdU^+^ cells decreased in the central retina from 48 hpf onwards and the proliferating region became restricted to the cilliary marginal zone at 72 hpf. However, in Mmp14a morphants, a high number of BrdU^+^ cells was still present in the central retina at 72 hpf (Figure 7D). Furthermore, quantitative analysis revealed that at 72 hpf the total number of BrdU^+^ cells was significantly higher in the morphants (BrdU^+^ cells/retinal section: 77±2 in morphants versus 45±2 in control embryos, *n* = 15 from 3 independent experiments, *p*<0.05). Of note, even at 120 hpf the proliferating region in the cilliary marginal zone of the morphant retinas was still larger as compared to control retinas (Figure 7D). Additional labeling experiments, using the chromatin binding fluorescent apoptotic marker acridine orange, did not reveal significant differences in the number and overall localization of apoptotic cells between morphant and control retinas at different developmental stages (Figure 7E).

**Figure 8:**
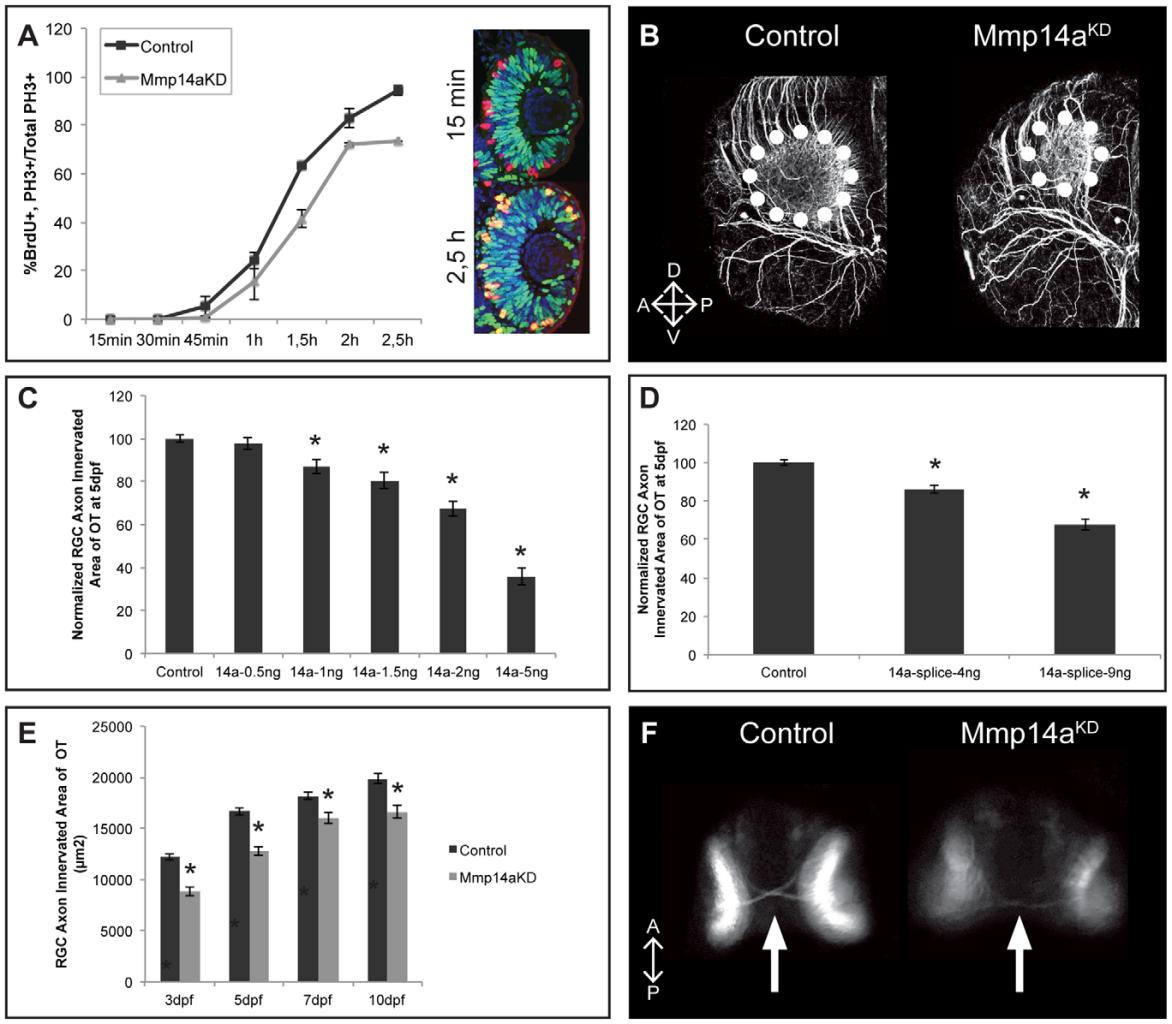
Mmp14a knockdown results in an impaired retinotectal development. **A** Graphical representation of the PLM from 15 minutes to 2.5 hours post-BrdU exposure at 31 hpf. The number of PH3^+^/BrdU^+^ retinoblasts is lower in retinas of Mmp14a morphant embryos as compared to control embryos, indicative for an impaired/prolonged transition from S to M phase at all time points examined (*n* = 10 from 2 independent experiments). Inset shows retinas of control embryos immunostained for BrdU (green) and PH3 staining (red) at 15 min and 2.5 h post-BrdU exposure. S-phase cells that have progressed from S to M phase are PH3^+^/BrdU^+^ (yellow). **B** Whole mount immunostaining for acetylated-α-Tubulin in 3 dpf embryos reveals a reduced tectal neuropil area (dotted circle), indicative for a reduced innervation of the OT by RGC axons. Images show a dorsolateral view of the left OT. **C–D** Quantitative analysis of the tectal area innervated by RGC axons reveals a dose-dependent decrease after Mmp14a knockdown using both the Mmp14a-ATG MO (**C**) and the Mmp14a-splice MO (**D**), as compared to control embryos at 5 dpf (*n* = 55 from 3 independent experiments). **E** Quantitative analysis of the tectal area innervated by RGC axons (in µm^2^) at different time-points between 3 and 10 dpf shows a significant and persistent reduction in RGC axon arborization area in Mmp14a morphant embryos as compared to controls (*n* = 55 from 3 independent experiments). **F** Knockdown of Mmp14a results in thinner optic nerves in 2 dpf transgenic *Tg(Ath5:GFP)* embryos, as compared to control embryos. Images show a ventral view on the optic chiasm (marked by white arrow). Data are represented as mean ± SEM (*p<0.05, Student's *t*-test for **C–D**, multilevel model statistical test (SAS proc mixed) for **A**, **E**). A, anterior; dpf: days post fertilization; D, dorsal; OT: optic tectum; PLM, percent labeled mitosis; P, posterior; RGC: retinal ganglion cells; V, ventral.

In order to further disentangle the mechanism underlying the delayed neurogenesis, we studied cell cycle kinetics in retinoblasts. To this end, we performed the percent labeled mitosis (PLM) paradigm test [33] in control and Mmp14a morphants at 31 hpf to determine the time needed for retinoblasts to proceed from S to M phase. The proportion of pH3^+^ cells that are also BrdU^+^ represent the PLM, and graphing this percentage over time provides an approximate readout of the duration between the S and M phases. This PLM test revealed a significant stalling of cells in the S phase in the Mmp14a morphant retinas. Indeed, while almost 100% of the retinoblasts progressed from S to M phase in control retinas at 2.5 h post BrdU exposure, only 73% of the Mmp14a morphant retinoblasts reached the M phase (Figure 8A).

Thus, Mmp14a knockdown clearly results in a prolonged retinoblast proliferation and a delayed retinal neurogenesis, but does not affect apoptosis.

### Mmp14a knockdown leads to a reduced axon innervation of the optic tectum

As immunofluorescent stainings showed Mmp14a expression in RGC nerve fibers and the optic nerve as well as in brain regions along the retinotectal path such as the POC and in the neuropil of the OT, we also evaluated retinotectal arborization by RGC axons after Mmp14a knockdown. Immunostaining for acetylated-α-Tubulin at 3 dpf revealed a highly significant reduction of the RGC axon innervation area in the OT of Mmp14a morphants as compared to control embryos (Figure 8B). Similarly, reduced RGC innervation of the tectal neuropil was also found in *Tg(Ath5:EGFP)* embryos. Dose-response experiments, using both the Mmp14a-ATG and the Mmp14a-splice MO, showed a dose-dependency of the observed phenotype (Figure 8C, 8D). Higher concentrations of the Mmp14a-ATG MO resulted in a more severe phenotype (Figure 8C). In fact, although morphant and control larvae grew substantially between 3 dpf and 5 dpf, innervation of the OT remained significantly smaller in the Mmp14a knockdown embryos until at least 10 dpf, despite similar body lengths (Figure 8E). Moreover, loss of Mmp14a in these transgenic fish also revealed a reduced thickness of the optic nerve at 48 hpf, possibly due to a reduced number of RGCs in the morphant retinas (Figure 8F).

In summary, knockdown of Mmp14a leads to embryos with a persistently impaired OT innervation.

### Mmp14b knockdown does not affect retinal or retinotectal pathway development

**Figure 9:**
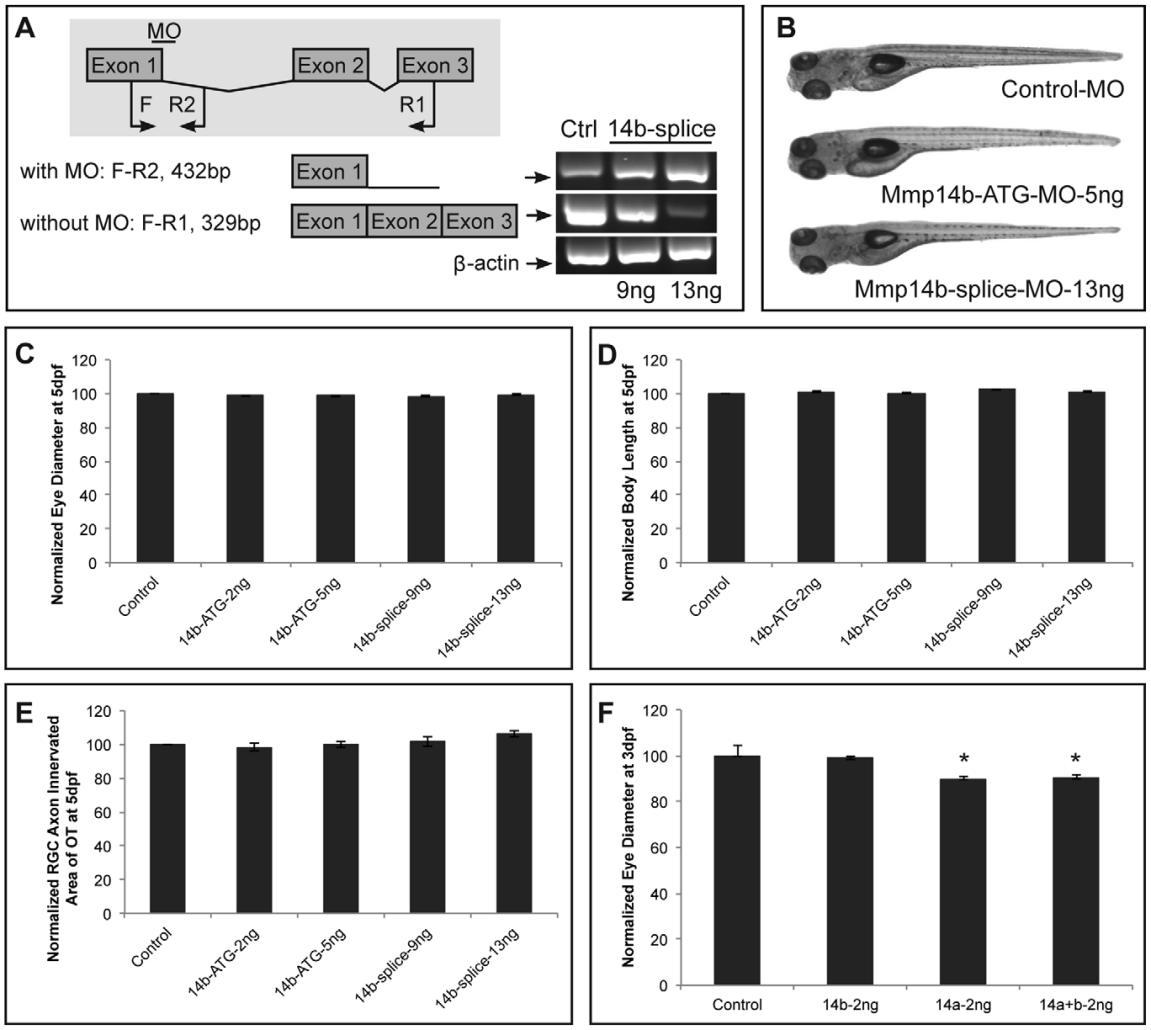
Mmp14b is not involved in retinal development. **A** RT-PCR analysis of Mmp14b-splice MO-injected embryos reveals efficient splice blocking, resulting in a dose-dependent increase in aberrantly spliced *mmp14b* mRNA. Since the splice MO targets intron 1 of *mmp14b*, which is 11 kb large, 2 different primer sets were used to visualize the splice blocking. Primer set F-R1 generates a fragment spanning exon 1 to 3 to detect correctly spliced transcripts (329 bp), and primer set F-R2 generates a fragment that spans exon 1 and part of intron 1 to detect aberrantly spliced transcripts (432 bp). The house keeping gene β-actin was used as a loading control. **B** Mmp14b knockdown using either the Mmp14b-ATG MO (5 ng) or the Mmp14b-splice MO (13 ng) in combination with the p53 MO does not affect normal embryonic morphogenesis at 5 dpf. **C–E** Quantitative analysis of eye size (**C**), total body length (**D**) and tectal area innervated by RGC axons (**E**) at 5 dpf, shows a normal eye diameter and tectal arborization area in Mmp14b morphant embryos using both the Mmp14b-ATG MO and Mmp14b-splice MO as compared to control embryos (*n* = 65 from 3 independent experiments)., **F** Combined knockdown of Mmp14a and Mmp14b (both injected at 2 ng) does not aggravate the eye defects observed after single Mmp14a knockdown in 3 dpf embryos (*n* = 70 from 3 independent experiments). Data are represented as mean ± SEM (*p<0.05, Student's *t*-test). Dpf, days post fertilization; F, forward; MO, morpholino; R, reverse.

Since zebrafish Mmp14 possesses two paralogs, *mmp14a* and *mmp14b*, similar analyses were performed after knockdown of Mmp14b, using a previously reported ATG-MO [28] or a splice-MO, both directed to *mmp14b*. The efficiency of the Mmp14b-splice MO was validated by RT-PCR (Figure 9A). Co-injections of the Mmp14b MOs with p53 MO resulted in embryos with a normal overall morphology (Figure 9B). Further analysis did not reveal significant differences in either eye or body size, when comparing Mmp14b- and control MO injected embryos at 5 dpf (Figure 9C, 9D). Furthermore, knockdown of Mmp14b with the ATG or splice MO did not affect proliferation, differentiation or OT innervation by RGC axons (data not shown and Figure 9E). Of note, double Mmp knockdown (combined injection with Mmp14a, Mmp14b and p53 MOs) did not potentiate the phenotype, as compared to single Mmp14a morphants (Figure 9F).

Therefore, Mmp14b does not appear to contribute to retinal neurogenesis or OT innervation.

### In vivo interaction between Mmp14a and Mmp2

**Figure 10:**
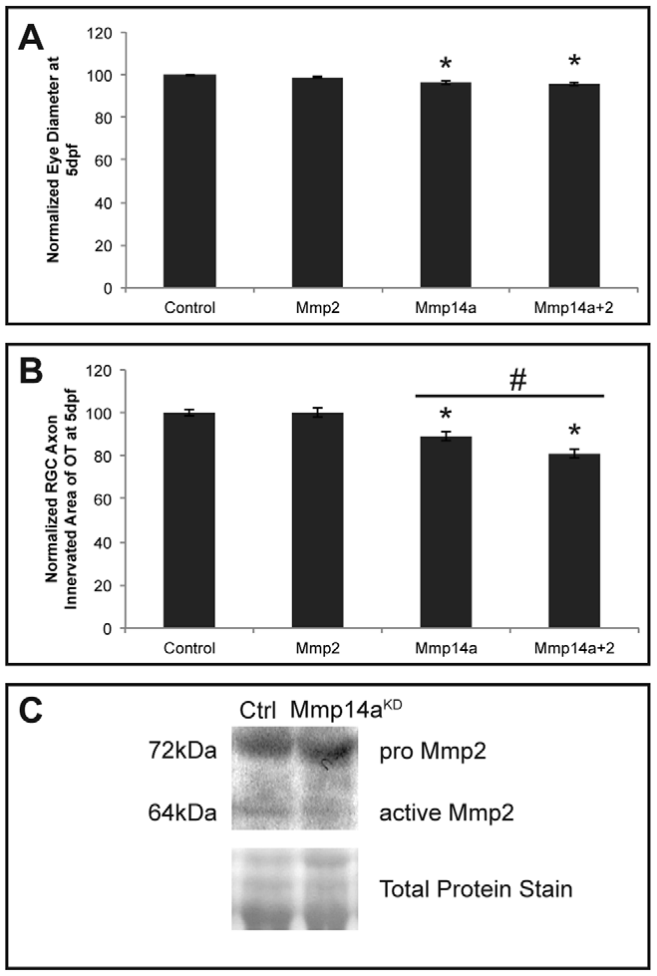
*In vivo* interaction between Mmp14a and Mmp2. **A, B** Quantitative analysis of eye size (**A**) and tectal area innervated by RGC axons (**B**) after single or combined suboptimal knockdown of Mmp14a and Mmp2 in 5 dpf embryos reveals that suboptimal knockdown of Mmp2 does not affect eye size nor OT innervation. However, combined knockdown of Mmp14a and Mmp2 results in embryos with normal eye size but a significantly reduced RGC axon innervation area in the OT, as compared to the Mmp14a suboptimal knockdown embryos (*n* = 75 from 3 independent experiments). **C** Western blot for Mmp2 on extracts of control and Mmp14a morphant embryos at 30 hpf shows reduced levels of active Mmp2 protein (64 kDa) in Mmp14a morphant embryos. Total protein coomassie blue staining was used for loading control. Data are represented as mean ± SEM (*p<0.05 versus controls using a Student's *t*-test; #*p*<0.05 between 1 ng injections of Mmp14a-MO and Mmp14a+Mmp2-MO using a multilevel model statistical test (SAS proc mixed)). dpf: days post fertilization; hpf: hours post fertilization; MO: morpholino; OT: optic tectum; RGC: retinal ganglion cells.

Mammalian MMP14 is known to act as a cell surface activator of pro-MMP2, thereby requiring assistance of tissue inhibitor of metalloproteinase 2 (TIMP2) [43]–[46]. Whether a similar interaction between these 2 MMPs also exists in lower vertebrates is currently unknown. Therefore, Mmp2 expression was first analyzed in comparison to Mmp14a expression. Whole mount ISH showed *mmp2* expression in the head region, including craniofacial elements, in myosepta and in the larger blood vessels of the tail in embryos from 24 hpf to 72 hpf (Figure S1A–C), consistent with previous reports [47], [48]. In addition, *mmp2* expression was also observed in the perichondria of the cartilage elements within the pharyngeal arches as well as in the connective tissue surrounding the brain, the nasal pit and the eye, comparable to the expression of *mmp14* (Figure S1B–G). On eye sections stained by ISH or IHC, Mmp2 mRNA and protein expression was found in the retina as early as 24 hpf, first in the retinoblast layer, and later on in the optic nerve and in the Müller glia fibers and their end feet at 72 hpf (Figure S1D–K). Within the brain, Mmp2-positive neurons were identified within the *stratum periventriculare* (SPV) layer of the OT, which sends fine projections towards the *stratum fibrosum et grisum superficiale* (SFGS) layer (Figure S1L). Overall, Mmp2 was clearly expressed in close proximity to Mmp14a in the eye and OT of developing zebrafish. Therefore, we continued to analyze the Mmp2 knockdown phenotype. A dose-response experiment was performed but an optimal MO concentration could not be obtained. Indeed, injections of low concentrations of the Mmp2 MO (1 ng, combined with p53 MO) did not affect embryo development nor eye size or RGC arborization in the OT (Figure S2A–D). However, injection of higher concentrations of Mmp2 MO (>1 ng, combined with p53 MO) resulted in embryos with a reduced OT innervation, eye size and body length, but these embryos also suffered from edema and other severe developmental defects, as compared to control embryos (Figure S2A–D). To investigate a potential co-involvement of Mmp2 in the phenotype observed in zebrafish after Mmp14a knockdown, combined Mmp14a and Mmp2 knockdown experiments were performed with submaximal doses for both ATG-MOs (both 1 ng, combined with p53 MO). Eye sizes were similarly reduced in double knockdown embryos as compared to single Mmp14a knockdown embryos (Figure 10A). However, the OT area, innervated by RGC axons, significantly decreased in double knockdown morphants as compared to embryos injected with 1 ng of Mmp14a-ATG MO only (Figure 10B). Notably, Western blot analysis of Mmp2 revealed reduced levels of active Mmp2 in Mmp14a knockdown embryos, indicating that also in zebrafish Mmp14a contributes to Mmp2 activation (Figure 10C).

Overall, these findings suggest a functional link between and a possible co-involvement of Mmp2 and Mmp14a in retinotectal development. Higher doses of Mmp2 MO disturb overall embryonic development, thereby precluding the investigation of its exact contribution to visual system development.

## Discussion

In this study we show that Mmp14a, but not Mmp14b, regulates retinal differentiation and retinotectal axon projection in the developing zebrafish. MMP14 was previously reported to be expressed in the retina of developing mouse eyes [19] and its expression has also been shown in the adult human, monkey and rodent eye in physiological as well as in pathological conditions [19], [49]–[53]. Although Mmps have already been linked to guidance of retinal axons in the developing visual system of *Xenopus* [13], [16], a specific involvement of MMP14 in retinotectal pathfinding and in retinogenesis remained elusive.

Consistent with previous reports, mRNA expression of *mmp14a* and *mmp14b* was found in the head region, including the craniofacial cartilage elements, and in the developing pectoral fins of zebrafish embryos [27], [28]. Furthermore, *mmp14a* and *b* expression was clearly detected in connective tissue/basement membrane of developing zebrafish embryos. Importantly, in-depth analysis of the ISH results and additional immunostainings also showed Mmp14a expression in the retina and in several brain regions from 24 hpf onwards with pronounced labeling in the INL, IPL and RGCL of the retina, in the optic nerve, the POC and the OT at 72 hpf. These findings correlate with the reported MMP14 expression in RGC and nerve fiber layers in the early postnatal mouse eye [19]. Strikingly, although *mmp14a* and *mmp14b* mRNA expression patterns overlap extensively in brain, cartilage and connective tissues, *mmp14b* expression levels in the eye are low at all time-points studied.

Knockdown using a MO designed against the ATG region of Mmp14a was previously reported to result in phenotypes with abnormal axis formation [28] or generated convergence and extension defects at the end of gastrulation [27]. Indeed, we observed similar axis formation defects when injecting the Mmp14a-ATG MO alone. However, in combination with a p53 MO, lower concentrations of a translation blocking or a splice Mmp14a MO did not result in the described phenotypes, nor did it affect normal embryonic or brain development, as revealed, respectively, by OVL analyses [20] and ISH for the neuronal markers *dlx2*, *pax2.1* and *shh*.

In contrast, our data unveiled that Mmp14a knockdown leads to microphthalmic eyes resulting from a retarded and attenuated retinal neurogenesis and a disrupted differentiation and lamination. One possible mechanism responsible for the reduced eye size and delayed retinal differentiation may be an altered progenitor cell proliferation. Indeed, Mmp14a morphant retinas showed an increased number of BrdU^+^ proliferating cells, as compared to control embryos. Since Mmp14a morphants show significantly reduced eyes in comparison to control embryos, it could be that an augmented or prolonged retinoblast proliferation is accompanied by an increased cell death [33]. However, acridine orange staining did not reveal significant differences in number of apoptotic cells in morphant retinas, as compared to control retinas. Another explanation for the observed microphthalmia, combined with an increase in proliferative cells, can be perturbed cell cycle kinetics, such that retinoblasts are unable to exit the cell cycle on time. A percent labeled mitosis (PLM) paradigm test, performed as described by Uribe and Gross [33], indeed revealed a significant delay of retinoblasts in moving from S to M phase in morphant retinas, as compared to controls. Therefore, we can conclude that the observed defects in retina development in the Mmp14a morphant embryos is at least partially due to a perturbed retinoblast cell cycle length. Nevertheless, and although neuronal differentiation seems to occur normal in the brain of Mmp14a morphant embryos, as shown via ISH for *dlx2*, *pax2.1* and *shh*, we can, at present, not exclude (in)direct effects of Mmp14a knockdown on neuronal differentiation in the retina. It is well known that both intrinsic, *e.g. ath5*, and extrinsic, *e.g. fgf*, *shh*, and *notch*, factors influence retinoblast proliferation, retinal neurogenesis and differentiation [54]–[58]. Whether Mmp14a affects any of these factors remains to be elucidated.

Knockdown of Mmp14a also caused a delayed arborization of the OT by RGC axons resulting in a reduced tectal innervation area, which was still obvious at 10 dpf. Previous findings in *Xenopus* larvae indeed revealed that RGC axons fail to make the caudal turn towards the OT when using the broad-spectrum MMP inhibitor GM6001 or to recognize the OT as their main target of innervation when administering the MMP2/9 specific inhibitor SB-3CT [13], [16]. Similarly, broad-spectrum and general Mmp inhibition perturbed tectal innervation in developing zebrafish. Although knockdown of Mmp14a alone did not prevent RGC axons to innervate the tectal neuropil, it significantly reduced the RGC axon arborization area. Of note, the disturbed innervation might be due to the observed defects in retinal neurogenesis induced by Mmp14a knockdown, in which case our data clearly show that a correct timing of the various developmental processes is necessary for the formation of a proper neural circuit. However, as Mmp14a is also expressed in the optic nerve, the retinotectal path and the OT, an additional direct or indirect effect of Mmp14a on axonal outgrowth/navigation cannot be excluded. Whether the observed abnormalities in tectal innervation have any functional consequences was not explored. Several tectum specific visual behavior tests could be performed, *i.e.* studying phototaxis, visual obstacle avoidance, visual escape response, prey capture and predator avoidance [59] to investigate possible visual deficits in Mmp14a morphants.

Mice deficient for MMP14 develop craniofacial dysmorphisms, dwarfism, osteopenia and arthritis due to loss of collagenolytic activity that is essential for modeling skeletal and extraskeletal connective tissues. As a result, MMP14 deficient mice die within 3 weeks after birth [38], [44]. Coyle *et al* also reported defects in craniofacial morphogenesis in developing zebrafish following Mmp14a knockdown [27]. Combination of lower doses of Mmp14a MOs and the p53 MO used in this study caused only mild defects in craniofacial cartilage development, again underlining the specificity of the retinal phenotype. Of note, MMP14 deficient mice were also reported to have a smaller body size and weight, but hair growth and eye opening occurred normally [38]. Since retinal neurogenesis has not yet been investigated in mice lacking MMP14, it remains elusive whether this proteinase affects eye and retinotectal development in higher vertebrates.

In contrast to Mmp14a, knockdown of Mmp14b did not result in any obvious defects in retinal neurogenesis or retinotectal pathway development. This finding is consistent with our ISH expression results, which showed abundant expression of *mmp14a* but only limited expression of *mmp14b* in the developing zebrafish eye. Moreover, it has already been reported that Mmp14a plays a more prominent role as compared to Mmp14b in various other developmental processes, including gastrulation movements and craniofacial cartilage development [27]. Overall, these findings identify Mmp14a as the predominant MMP14 paralog involved in zebrafish retinogenesis.

The best-described proteolytic function of mammalian MMP14 is its action as a cell surface activator of pro-MMP2, which requires the assistance of tissue inhibitor of metalloproteinase 2 (TIMP2). Pro-MMP2 forms a tight complex with TIMP2 that binds with its N-terminal inhibitory domain to MMP14 on the cell surface. Activation of pro-MMP2 takes place when another adjacent ‘free’ MMP14 unit proteolytically removes the propeptide of pro-MMP2 in this trimeric MMP14-TIMP2-pro-MMP2 complex [43]–[45]. Therefore, we also investigated Mmp2 expression and loss-of-function in more detail in in developing zebrafish. Zhang *et al* described diffuse *mmp2* expression along the entire anterior to posterior axis of embryos up to 72 hpf [60], while Hillegass *et al* also reported a more specific *mmp2* expression in the neurocranial cartilage and midbrain of 72 hpf zebrafish embryos [61]. Several groups showed *mmp2* expression in the blood vessels in the tail, *i.e.* in the dorsal aorta, posterior cardinal vein, intersomitic vessels and dorsal longitudinal anastomotic vessel [47], [48], [62]. Using whole mount ISH, we also found *mmp2* expression in the head region, in craniofacial cartilage elements and in the blood vessels in the trunk. Furthermore, *mmp2* expression was also present in connective tissue of developing zebrafish embryos, as was observed for *mmp14a* and *b*. Strikingly, additional immunostaining revealed Mmp2 expression in the Müller glia fibers and their end feet within the retina. These findings are in line with a previous report where MMP2 was found to be expressed in the INL of the mouse retina at early postnatal stages [19]. Additional staining was also observed in the optic nerve head and in the OT. In the OT, Mmp2 was expressed by a specific population of neurons, lying within the *stratum periventriculare* (SPV) and specifically projecting towards the *stratum fibrosum et grisum superficiale* (SFGS), which identifies these Mmp2-positive neurons as periventricular interneurons [59], [63].

Mmp2 knockdown in zebrafish has been reported to result in several developmental abnormalities [60]. Using high doses of Mmp2 MO, we indeed obtained embryos with shortened body axis, edema and several other developmental defects. However, lower doses of Mmp2 MO resulted in zebrafish embryos with an overall normal morphology, including normal eye size. These data suggest that Mmp2 plays an important role in embryogenesis and therefore its role in visual system development cannot be investigated using general Mmp2 knockdown. In contrast, MMP2-deficient mice, although reported to be smaller at birth, lack a severe phenotype [64], [65]. However, MMP2-MMP14 double deficient mice die at birth, indicating a functional overlap between these 2 proteinases in mice. Of note, in MMP14-null mice, MMP2 activity is reduced, but not eliminated, while pro-MMP2 levels are increased [66]. Similarly, our Western blot results revealed reduced Mmp2 activity levels and increased levels of pro-Mmp2 in Mmp14a knockdown embryos, suggesting Mmp14a as a major activator of pro-Mmp2 *in vivo*. Furthermore, double knockdown of Mmp2 and Mmp14a in zebrafish resulted in a significantly aggravated RGC axonal arborization defect of the OT, as compared to submaximal single knockdown of Mmp14a, suggestive for a functional link between Mmp2 and Mmp14a in retinotectal development in zebrafish. Although previously published data show co-expression of *mmp14*, *mmp2* and *timp2* mRNA transcripts in regenerating caudal fins in zebrafish [67] and Zhang *et al* showed the importance of Mmp14, Mmp2 and Timp2 for normal zebrafish development ([28], [46], [60], we hereby identify for the first time a potential *in vivo* interaction between Mmp14a and Mmp2 in zebrafish.

In summary, our findings reveal that the membrane-bound metalloproteinase Mmp14a modulates retinoblast cell cycle kinetics, and thereby influences retinal neurogenesis, differentiation, lamination as well as proper retinotectal axon projection and tectal innervation.

## Supporting Information

Figure S1 **Spatiotemporal expression pattern of Mmp2 in developing zebrafish.** **A–C** Whole mount *in situ* hybridization (ISH) for *mmp2* in zebrafish embryos at various developmental stages shows *mmp2* mRNA expression in the head, in myosepta (black arrow in **A**–**C**) and major blood vessels (grey arrow in **A**–**C**) in the tail at 24, 48 and 72 hpf. *Mmp2* expressing craniofacial elements (white arrow in **B** and **C**) are visible from 48 hpf onwards. **D–G** Transverse sections through the head of zebrafish embryos, stained via whole mount ISH for *mmp2* and counterstained with Nuclear Fast Red, show *mmp2* expression in the brain and retina from 24 hpf onwards, in connective tissue (white arrow) from 32 hpf and in cartilage (black arrow) at 48 and 72 hpf. **H–L** Immunohistochemical stainings on transverse sections, made through the head of zebrafish embryos of various developmental stages, reveal Mmp2 protein expression (red) in the retina and the brain from 32 hpf onwards. Connective tissue and cartilage is labeled at 48 and 72 hpf. A higher magnification of the eye shows Mmp2 expression in Müller glia (white arrow) and their end feet and in the optic nerve head (white arrowhead) (**K**). A detailed view of the OT reveals Mmp2 expression in interneurons in the SPV and in their connections in the SFGS layer of the tectal neuropil (**L**). DAPI (blue) was used as counterstain. hpf, hours post fertilization; OT, optic tectum; SFGS, stratum fibrosum et grisum superficiale; SPV, stratum periventriculare. Scale bars: 50 µm, except panel L: 100 µm.
(TIF)

Figure S2 **Mmp2 knockdown results in embryos with severe developmental defects.** **A** Knockdown of Mmp2, obtained after injection of 1 ng of both Mmp2-ATG and p53 MOs, does not affect normal embryonic morphogenesis in Mmp2 morphants at 3 dpf, as compared to control embryos. However, embryos injected with 2.5 ng–4 ng of both Mmp2-ATG and p53 MOs show edema and a deformed body axis. **B–D** Analysis of eye size (**B**), total body length (**C**), and tectal area innervated by RGC axons (**D**) reveals a significant and dose-dependent decrease in eye size, tectal innervation area but also body length, after knockdown of Mmp2 with the ATG MO in 5 dpf morphants, as compared to control embryos (*n* = 45 from 2 independent experiments). A MO concentration of 1 ng does not affect any of these parameters and results in normally developing embryos. Eye size, body length and tectal innervation area are normalized towards average values in control embryos. Data are represented as mean ± SEM (*p-value<0.05, Student's *t*-test). dpf, days post fertilization; MO, morpholino; OT, optic tectum; RGC, retinal ganglion cell.
(TIF)

## References

[1] Paridaen JT, Janson E, Utami KH, Pereboom TC, Essers PB, et al (2011) The nucleolar GTP-binding proteins Gnl2 and nucleostemin are required for retinal neurogenesis in developing zebrafish. Dev Biol 355: 286–301. 21565180 10.1016/j.ydbio.2011.04.028

[2] Haupt C, Huber AB (2008) How axons see their way–axonal guidance in the visual system. Front Biosci 13: 3136–3149. 17981783 10.2741/2915

[3] Glass AS, Dahm R (2004) The zebrafish as a model organism for eye development. Ophthalmic Res 36: 4–24. 15007235 10.1159/000076105

[4] Renninger SL, Schonthaler HB, Neuhauss SC, Dahm R (2011) Investigating the genetics of visual processing, function and behaviour in zebrafish. Neurogenetics 12: 97–116. 21267617 10.1007/s10048-011-0273-x

[5] Schmitt EA, Dowling JE (1999) Early retinal development in the zebrafish, Danio rerio: light and electron microscopic analyses. J Comp Neurol 404: 515–536. 9987995

[6] Zhao XC, Yee RW, Norcom E, Burgess H, Avanesov AS, et al (2006) The zebrafish cornea: structure and development. Invest Ophthalmol Vis Sci 47: 4341–4348. 17003424 10.1167/iovs.05-1611

[7] Stenkamp DL (2007) Neurogenesis in the fish retina. Int Rev Cytol 259: 173–224. 17425942 10.1016/S0074-7696(06)59005-9 PMC2897061

[8] Malicki J, Jo H, Wei X, Hsiung M, Pujic Z (2002) Analysis of gene function in the zebrafish retina. Methods 28: 427–438. 12507461 10.1016/s1046-2023(02)00262-1

[9] Avanesov A, Malicki J (2004) Approaches to study neurogenesis in the zebrafish retina. Methods Cell Biol 76: 333–384. 15602883 10.1016/s0091-679x(04)76016-1

[10] Stuermer CA (1988) Retinotopic organization of the developing retinotectal projection in the zebrafish embryo. J Neurosci 8: 4513–4530. 2848935 10.1523/JNEUROSCI.08-12-04513.1988 PMC6569580

[11] Karlstrom RO, Trowe T, Klostermann S, Baier H, Brand M, et al (1996) Zebrafish mutations affecting retinotectal axon pathfinding. Development 123: 427–438. 9007260 10.1242/dev.123.1.427

[12] Page-McCaw A, Ewald AJ, Werb Z (2007) Matrix metalloproteinases and the regulation of tissue remodelling. Nat Rev Mol Cell Biol 8: 221–233. 17318226 10.1038/nrm2125 PMC2760082

[13] Hehr CL, Hocking JC, McFarlane S (2005) Matrix metalloproteinases are required for retinal ganglion cell axon guidance at select decision points. Development 132: 3371–3379. 15975939 10.1242/dev.01908

[14] Rivera S, Khrestchatisky M, Kaczmarek L, Rosenberg GA, Jaworski DM (2010) Metzincin proteases and their inhibitors: foes or friends in nervous system physiology? J Neurosci 30: 15337–15357. 21084591 10.1523/JNEUROSCI.3467-10.2010 PMC3072038

[15] Zhang H, Adwanikar H, Werb Z, Noble-Haeusslein LJ (2010) Matrix metalloproteinases and neurotrauma: evolving roles in injury and reparative processes. Neuroscientist 16: 156–170. 20400713 10.1177/1073858409355830 PMC2858362

[16] Webber CA, Hocking JC, Yong VW, Stange CL, McFarlane S (2002) Metalloproteases and guidance of retinal axons in the developing visual system. J Neurosci 22: 8091–8100. 12223563 10.1523/JNEUROSCI.22-18-08091.2002 PMC6758082

[17] Llano E, Adam G, Pendas AM, Quesada V, Sanchez LM, et al (2002) Structural and enzymatic characterization of Drosophila Dm2-MMP, a membrane-bound matrix metalloproteinase with tissue-specific expression. J Biol Chem 277: 23321–23329. 11967260 10.1074/jbc.M200121200

[18] Huh MI, Lee YM, Seo SK, Kang BS, Chang Y, et al (2007) Roles of MMP/TIMP in regulating matrix swelling and cell migration during chick corneal development. J Cell Biochem 101: 1222–1237. 17295208 10.1002/jcb.21246

[19] Gariano RF, Hu D, Helms J (2006) Expression of angiogenesis-related genes during retinal development. Gene Expr Patterns 6: 187–192. 16330258 10.1016/j.modgep.2005.06.008

[20] Kimmel CB, Ballard WW, Kimmel SR, Ullmann B, Schilling TF (1995) Stages of embryonic development of the zebrafish. Dev Dyn 203: 253–310. 8589427 10.1002/aja.1002030302

[21] Kay JN, Link BA, Baier H (2005) Staggered cell-intrinsic timing of ath5 expression underlies the wave of ganglion cell neurogenesis in the zebrafish retina. Development 132: 2573–2585. 15857917 10.1242/dev.01831

[22] Thisse C, Thisse B (2008) High-resolution in situ hybridization to whole-mount zebrafish embryos. Nat Protoc 3: 59–69. 18193022 10.1038/nprot.2007.514

[23] Li Y, Allende ML, Finkelstein R, Weinberg ES (1994) Expression of two zebrafish orthodenticle-related genes in the embryonic brain. Mech Dev 48: 229–244. 7893604 10.1016/0925-4773(94)90062-0

[24] Schilling TF, Kimmel CB (1994) Segment and cell type lineage restrictions during pharyngeal arch development in the zebrafish embryo. Development 120: 483–494. 8162849 10.1242/dev.120.3.483

[25] Uribe RA, Gross JM (2007) Immunohistochemistry on cryosections from embryonic and adult zebrafish eyes. CSH Protoc 2007: pdb prot4779. 21357120 10.1101/pdb.prot4779

[26] Hughes DP, Andersen SB, Hywel-Jones NL, Himaman W, Billen J, et al (2011) Behavioral mechanisms and morphological symptoms of zombie ants dying from fungal infection. BMC Ecol 11: 13. 21554670 10.1186/1472-6785-11-13 PMC3118224

[27] Coyle RC, Latimer A, Jessen JR (2008) Membrane-type 1 matrix metalloproteinase regulates cell migration during zebrafish gastrulation: evidence for an interaction with non-canonical Wnt signaling. Exp Cell Res 314: 2150–2162. 18423448 10.1016/j.yexcr.2008.03.010

[28] Zhang J, Bai S, Zhang X, Nagase H, Sarras MP Jr (2003) The expression of novel membrane-type matrix metalloproteinase isoforms is required for normal development of zebrafish embryos. Matrix Biol 22: 279–293. 12853038 10.1016/s0945-053x(03)00020-9

[29] Robu ME, Larson JD, Nasevicius A, Beiraghi S, Brenner C, et al (2007) p53 activation by knockdown technologies. PLoS Genet 3: e78. 17530925 10.1371/journal.pgen.0030078 PMC1877875

[30] Van Hove I, Verslegers M, Buyens T, Delorme N, Lemmens K, et al (2012) An aberrant cerebellar development in mice lacking matrix metalloproteinase-3. Mol Neurobiol 45: 17–29. 22108898 10.1007/s12035-011-8215-z

[31] Welinder C, Ekblad L (2011) Coomassie staining as loading control in Western blot analysis. J Proteome Res 10: 1416–1419. 21186791 10.1021/pr1011476

[32] Walpita CN, Crawford AD, Janssens ED, Van der Geyten S, Darras VM (2009) Type 2 iodothyronine deiodinase is essential for thyroid hormone-dependent embryonic development and pigmentation in zebrafish. Endocrinology 150: 530–539. 18801906 10.1210/en.2008-0457

[33] Uribe RA, Gross JM (2010) Id2a influences neuron and glia formation in the zebrafish retina by modulating retinoblast cell cycle kinetics. Development 137: 3763–3774. 20943708 10.1242/dev.050484 PMC3049276

[34] Erickson T, French CR, Waskiewicz AJ (2010) Meis1 specifies positional information in the retina and tectum to organize the zebrafish visual system. Neural Dev 5: 22. 20809932 10.1186/1749-8104-5-22 PMC2939508

[35] Diekmann H, Stuermer CA (2009) Zebrafish neurolin-a and -b, orthologs of ALCAM, are involved in retinal ganglion cell differentiation and retinal axon pathfinding. J Comp Neurol 513: 38–50. 19107846 10.1002/cne.21928

[36] Akimenko MA, Ekker M, Wegner J, Lin W, Westerfield M (1994) Combinatorial expression of three zebrafish genes related to distal-less: part of a homeobox gene code for the head. J Neurosci 14: 3475–3486. 7911517 10.1523/JNEUROSCI.14-06-03475.1994 PMC6576961

[37] Nakada C, Satoh S, Tabata Y, Arai K, Watanabe S (2006) Transcriptional repressor foxl1 regulates central nervous system development by suppressing shh expression in zebra fish. Mol Cell Biol 26: 7246–7257. 16980626 10.1128/MCB.00429-06 PMC1592895

[38] Holmbeck K, Bianco P, Caterina J, Yamada S, Kromer M, et al (1999) MT1-MMP-deficient mice develop dwarfism, osteopenia, arthritis, and connective tissue disease due to inadequate collagen turnover. Cell 99: 81–92. 10520996 10.1016/s0092-8674(00)80064-1

[39] Bovolenta P, Mallamaci A, Briata P, Corte G, Boncinelli E (1997) Implication of OTX2 in pigment epithelium determination and neural retina differentiation. J Neurosci 17: 4243–4252. 9151741 10.1523/JNEUROSCI.17-11-04243.1997 PMC6573571

[40] Hever AM, Williamson KA, van Heyningen V (2006) Developmental malformations of the eye: the role of PAX6, SOX2 and OTX2. Clin Genet 69: 459–470. 16712695 10.1111/j.1399-0004.2006.00619.x

[41] Liu Y, Shen Y, Rest JS, Raymond PA, Zack DJ (2001) Isolation and characterization of a zebrafish homologue of the cone rod homeobox gene. Invest Ophthalmol Vis Sci 42: 481–487. 11157887

[42] Easter SS Jr, Malicki JJ (2002) The zebrafish eye: developmental and genetic analysis. Results Probl Cell Differ 40: 346–370. 12353485 10.1007/978-3-540-46041-1_17

[43] Visse R, Nagase H (2003) Matrix metalloproteinases and tissue inhibitors of metalloproteinases: structure, function, and biochemistry. Circ Res 92: 827–839. 12730128 10.1161/01.RES.0000070112.80711.3D

[44] Klein T, Bischoff R (2011) Physiology and pathophysiology of matrix metalloproteases. Amino Acids 41: 271–290. 20640864 10.1007/s00726-010-0689-x PMC3102199

[45] Sternlicht MD, Werb Z (2001) How matrix metalloproteinases regulate cell behavior. Annu Rev Cell Dev Biol 17: 463–516. 11687497 10.1146/annurev.cellbio.17.1.463 PMC2792593

[46] Zhang J, Bai S, Tanase C, Nagase H, Sarras MP Jr (2003) The expression of tissue inhibitor of metalloproteinase 2 (TIMP-2) is required for normal development of zebrafish embryos. Dev Genes Evol 213: 382–389. 12736828 10.1007/s00427-003-0333-9

[47] Serluca FC, Drummond IA, Fishman MC (2002) Endothelial signaling in kidney morphogenesis: a role for hemodynamic forces. Curr Biol 12: 492–497. 11909536 10.1016/s0960-9822(02)00694-2

[48] Keow JY, Pond ED, Cisar JS, Cravatt BF, Crawford BD (2012) Activity-based labeling of matrix metalloproteinases in living vertebrate embryos. PLoS One 7: e43434. 22952682 10.1371/journal.pone.0043434 PMC3429480

[49] Smine A, Plantner JJ (1997) Membrane type-1 matrix metalloproteinase in human ocular tissues. Curr Eye Res 16: 925–929. 9288454 10.1076/ceyr.16.9.925.5044

[50] Sivak JM, Fini ME (2002) MMPs in the eye: emerging roles for matrix metalloproteinases in ocular physiology. Prog Retin Eye Res 21: 1–14. 11906808 10.1016/s1350-9462(01)00015-5

[51] Agapova OA, Kaufman PL, Lucarelli MJ, Gabelt BT, Hernandez MR (2003) Differential expression of matrix metalloproteinases in monkey eyes with experimental glaucoma or optic nerve transection. Brain Res 967: 132–143. 12650974 10.1016/s0006-8993(02)04234-8

[52] Ahmed F, Brown KM, Stephan DA, Morrison JC, Johnson EC, et al (2004) Microarray analysis of changes in mRNA levels in the rat retina after experimental elevation of intraocular pressure. Invest Ophthalmol Vis Sci 45: 1247–1258. 15037594 10.1167/iovs.03-1123

[53] Tucker B, Klassen H, Yang L, Chen DF, Young MJ (2008) Elevated MMP Expression in the MRL Mouse Retina Creates a Permissive Environment for Retinal Regeneration. Invest Ophthalmol Vis Sci 49: 1686–1695. 18385092 10.1167/iovs.07-1058 PMC2613950

[54] Agathocleous M, Harris WA (2009) From progenitors to differentiated cells in the vertebrate retina. Annu Rev Cell Dev Biol 25: 45–69. 19575661 10.1146/annurev.cellbio.042308.113259

[55] Vinothkumar S, Rastegar S, Takamiya M, Ertzer R, Strahle U (2008) Sequential and cooperative action of Fgfs and Shh in the zebrafish retina. Dev Biol 314: 200–214. 18177854 10.1016/j.ydbio.2007.11.034

[56] Neumann CJ, Nuesslein-Volhard C (2000) Patterning of the zebrafish retina by a wave of sonic hedgehog activity. Science 289: 2137–2139. 11000118 10.1126/science.289.5487.2137

[57] Martinez-Morales JR, Del Bene F, Nica G, Hammerschmidt M, Bovolenta P, et al (2005) Differentiation of the vertebrate retina is coordinated by an FGF signaling center. Dev Cell 8: 565–574. 15809038 10.1016/j.devcel.2005.01.022

[58] Nakayama Y, Miyake A, Nakagawa Y, Mido T, Yoshikawa M, et al (2008) Fgf19 is required for zebrafish lens and retina development. Dev Biol 313: 752–766. 18089288 10.1016/j.ydbio.2007.11.013

[59] Nevin LM, Robles E, Baier H, Scott EK (2010) Focusing on optic tectum circuitry through the lens of genetics. BMC Biol 8: 126. 20920150 10.1186/1741-7007-8-126 PMC2949621

[60] Zhang J, Bai S, Zhang X, Nagase H, Sarras MP Jr (2003) The expression of gelatinase A (MMP-2) is required for normal development of zebrafish embryos. Dev Genes Evol 213: 456–463. 12898250 10.1007/s00427-003-0346-4

[61] Hillegass JM, Villano CM, Cooper KR, White LA (2008) Glucocorticoids alter craniofacial development and increase expression and activity of matrix metalloproteinases in developing zebrafish (Danio rerio). Toxicol Sci 102: 413–424. 18281261 10.1093/toxsci/kfn010

[62] Detry B, Erpicum C, Paupert J, Blacher S, Maillard C, et al (2012) Matrix metalloproteinase-2 governs lymphatic vessel formation as an interstitial collagenase. Blood 10.1182/blood-2011-12-400267 22490679

[63] Robles E, Smith SJ, Baier H (2011) Characterization of genetically targeted neuron types in the zebrafish optic tectum. Front Neural Circuits 5: 1. 21390291 10.3389/fncir.2011.00001 PMC3046383

[64] Itoh T, Ikeda T, Gomi H, Nakao S, Suzuki T, et al (1997) Unaltered secretion of beta-amyloid precursor protein in gelatinase A (matrix metalloproteinase 2)-deficient mice. J Biol Chem 272: 22389–22392. 9278386 10.1074/jbc.272.36.22389

[65] Mosig RA, Dowling O, DiFeo A, Ramirez MC, Parker IC, et al (2007) Loss of MMP-2 disrupts skeletal and craniofacial development and results in decreased bone mineralization, joint erosion and defects in osteoblast and osteoclast growth. Hum Mol Genet 16: 1113–1123. 17400654 10.1093/hmg/ddm060 PMC2576517

[66] Zhou Z, Apte SS, Soininen R, Cao R, Baaklini GY, et al (2000) Impaired endochondral ossification and angiogenesis in mice deficient in membrane-type matrix metalloproteinase I. Proc Natl Acad Sci U S A 97: 4052–4057. 10737763 10.1073/pnas.060037197 PMC18145

[67] Bai S, Thummel R, Godwin AR, Nagase H, Itoh Y, et al (2005) Matrix metalloproteinase expression and function during fin regeneration in zebrafish: analysis of MT1-MMP, MMP2 and TIMP2. Matrix Biol 24: 247–260. 15935631 10.1016/j.matbio.2005.03.007

