# Supplementary figures and images for: Matrix Metalloproteinase 14 in the Zebrafish: An Eye on Retinal and Retinotectal Development
Source: PLoS One. 2013 Jan 9;8(1):e52915. doi: 10.1371/journal.pone.0052915 (PMC3541391; doi:10.1371/journal.pone.0052915)

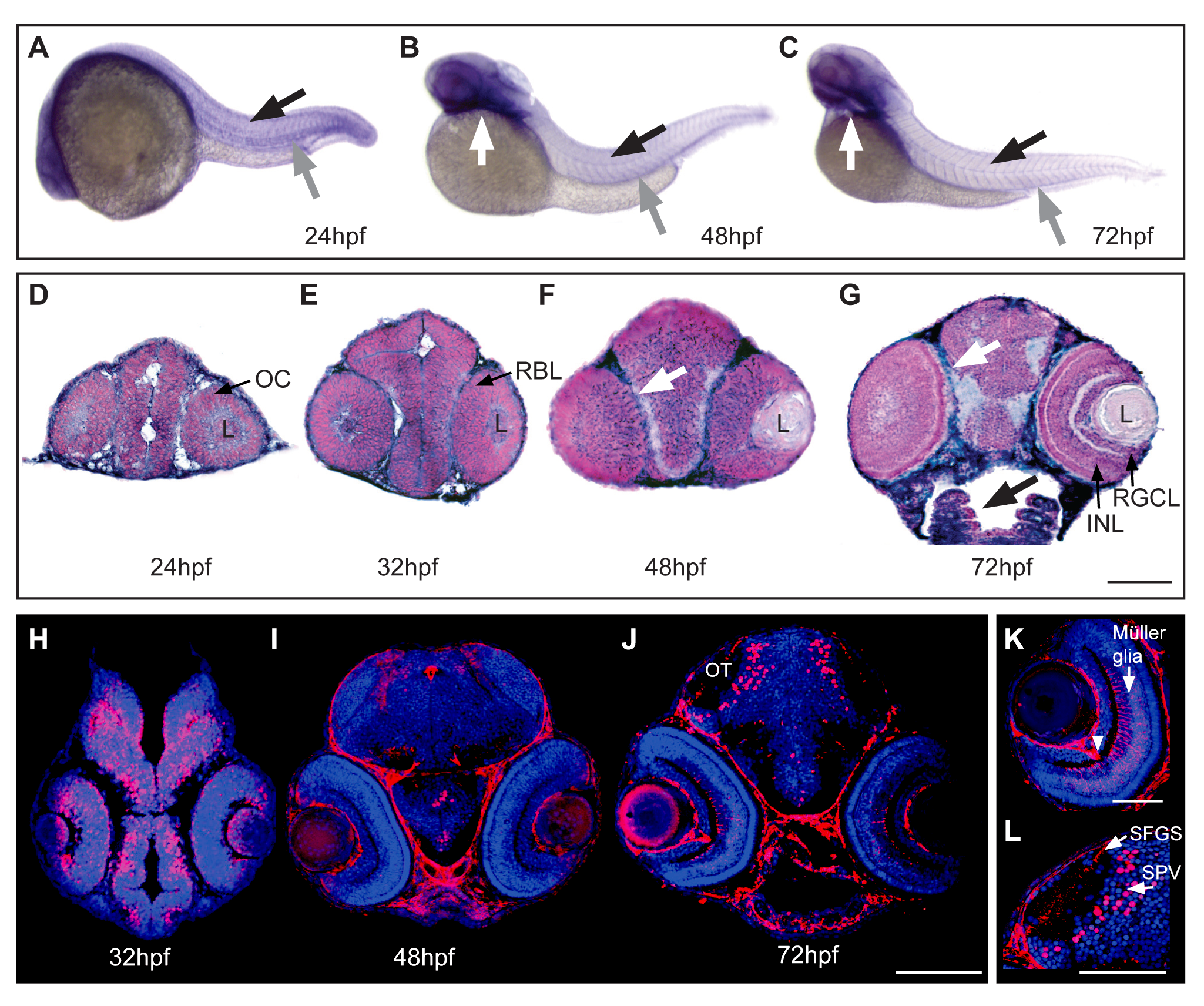

Supplement: Figure S1 — Spatiotemporal expression pattern of Mmp2 in developing zebrafish. A–C Whole mount in situ hybridization (ISH) for mmp2 in zebrafish embryos at various developmental stages shows mmp2 mRNA expression in the head, in myosepta (black arrow in A–C) and major blood vessels (grey arrow in A–C) in the tail at 24, 48 and 72 hpf. Mmp2 expressing craniofacial elements (white arrow in B and C) are visible from 48 hpf onwards. D–G Transverse sections through the head of zebrafish embryos, stained via whole mount ISH for mmp2 and counterstained with Nuclear Fast Red, show mmp2 expression in the brain and retina from 24 hpf onwards, in connective tissue (white arrow) from 32 hpf and in cartilage (black arrow) at 48 and 72 hpf. H–L Immunohistochemical stainings on transverse sections, made through the head of zebrafish embryos of various developmental stages, reveal Mmp2 protein expression (red) in the retina and the brain from 32 hpf onwards. Connective tissue and cartilage is labeled at 48 and 72 hpf. A higher magnification of the eye shows Mmp2 expression in Müller glia (white arrow) and their end feet and in the optic nerve head (white arrowhead) (K). A detailed view of the OT reveals Mmp2 expression in interneurons in the SPV and in their connections in the SFGS layer of the tectal neuropil (L). DAPI (blue) was used as counterstain. hpf, hours post fertilization; OT, optic tectum; SFGS, stratum fibrosum et grisum superficiale; SPV, stratum periventriculare. Scale bars: 50 µm, except panel L: 100 µm. (TIF) [file pone.0052915.s001.tif]

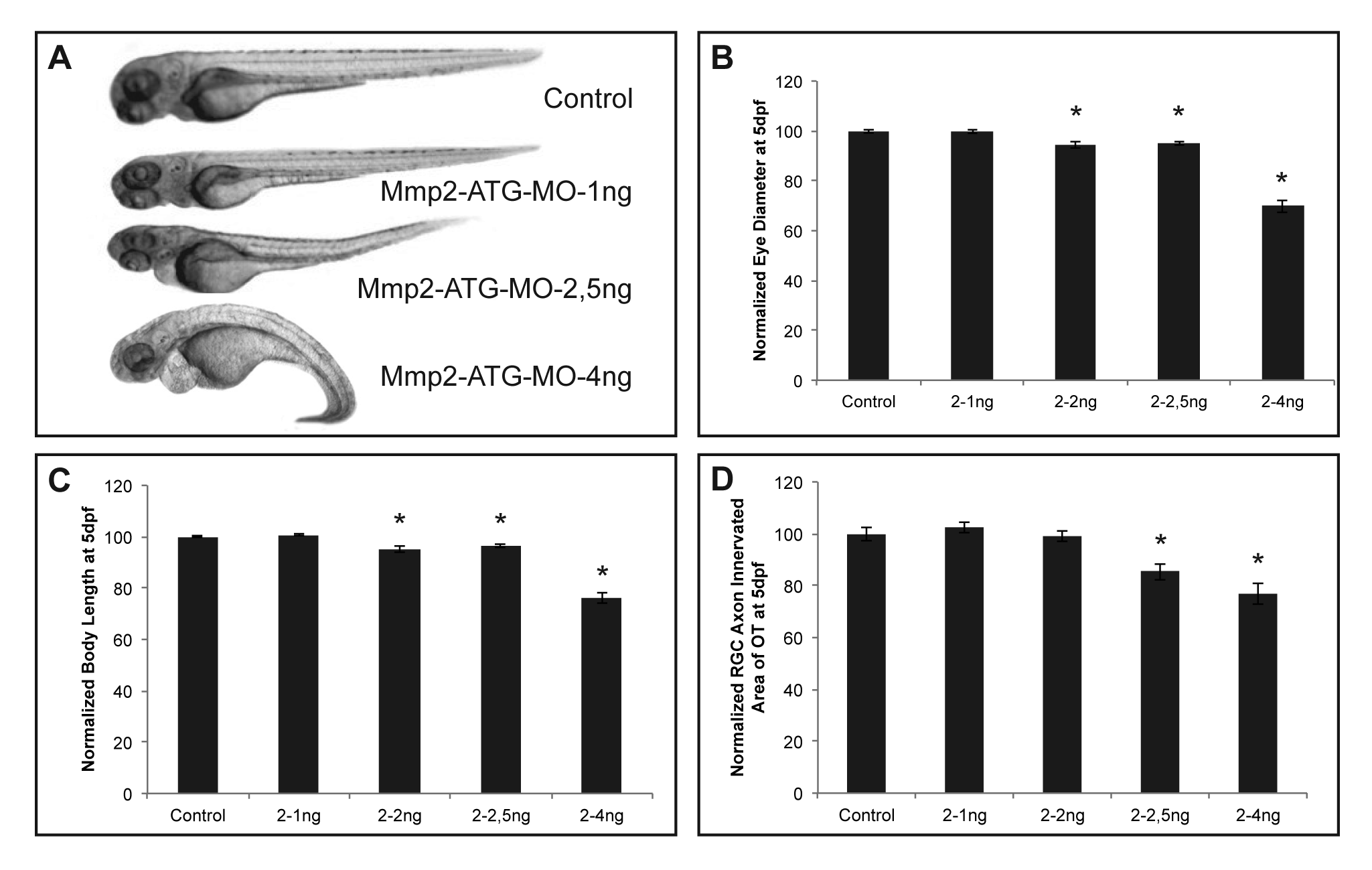

Supplement: Figure S2 — Mmp2 knockdown results in embryos with severe developmental defects. A Knockdown of Mmp2, obtained after injection of 1 ng of both Mmp2-ATG and p53 MOs, does not affect normal embryonic morphogenesis in Mmp2 morphants at 3 dpf, as compared to control embryos. However, embryos injected with 2.5 ng–4 ng of both Mmp2-ATG and p53 MOs show edema and a deformed body axis. B–D Analysis of eye size (B), total body length (C), and tectal area innervated by RGC axons (D) reveals a significant and dose-dependent decrease in eye size, tectal innervation area but also body length, after knockdown of Mmp2 with the ATG MO in 5 dpf morphants, as compared to control embryos (n = 45 from 2 independent experiments). A MO concentration of 1 ng does not affect any of these parameters and results in normally developing embryos. Eye size, body length and tectal innervation area are normalized towards average values in control embryos. Data are represented as mean ± SEM (*p-value<0.05, Student's t-test). dpf, days post fertilization; MO, morpholino; OT, optic tectum; RGC, retinal ganglion cell. (TIF) [file pone.0052915.s002.tif]
